# Cardiovascular magnetic resonance artefacts

**DOI:** 10.1186/1532-429X-15-41

**Published:** 2013-05-22

**Authors:** Pedro F Ferreira, Peter D Gatehouse, Raad H Mohiaddin, David N Firmin

**Affiliations:** 1 National Heart and Lung Institute, Imperial College, London, UK; 2 Royal Brompton Hospital, London, UK

## Abstract

The multitude of applications offered by CMR make it an increasing popular modality to study the heart and the surrounding vessels. Nevertheless the anatomical complexity of the chest, together with cardiac and respiratory motion, and the fast flowing blood, present many challenges which can possibly translate into imaging artefacts. The literature is wide in terms of papers describing specific MR artefacts in great technical detail. In this review we attempt to summarise, in a language accessible to a clinical readership, some of the most common artefacts found in CMR applications. It begins with an introduction of the most common pulse sequences, and imaging techniques, followed by a brief section on typical cardiovascular applications. This leads to the main section on common CMR artefacts with examples, a short description of the mechanisms behind them, and possible solutions.

## Review

### Introduction

There are unique motion and other issues involved in Cardiovascular Magnetic Resonance (CMR) that can lead to artefacts which can obscure or easily be misinterpreted as pathology. An artefact can be defined as something that is visible in an image but it is artificial, and is often detrimental to diagnosis. For this reason it is important to have an understanding of the physical principles behind the formation of such artefacts so that they can be identified and possibly avoided.

A large range of different sequences are used for the different applications in CMR and the majority of these are still being developed to improve their accuracy and reliability. It is therefore impossible to be completely comprehensive covering all artefacts for all sequences, thus the examples of artefact have been restricted to one or two of the most problematic or common. The artefacts are also specific to cardiovascular imaging, and more general artefacts related to hardware are omitted.

This article, that is designed for a clinical readership, follows previous publications on the subject [1-3]. There is initially a brief description of the most common sequences and preparation pulses used, along with imaging techniques and typical cardiovascular applications. The main section follows, with common examples of artefacts, accompanied with a small description of the mechanisms behind them and possible solutions and trade-offs. At this point it is quite possible that some readers will not require the introductory sections describing cardiovascular pulse sequences and applications and would happily move directly to the main sections describing artefacts. If this is the case then please jump to section *Cardiovascular Magnetic Resonance Artefacts*. Also, alternatives to these sections are available elsewhere such as the excellent physics articles from Ridgway and Biglands in JCMR 2010 and 2012 [4,5].

### Cardiovascular pulse sequences

**Figure 1:**
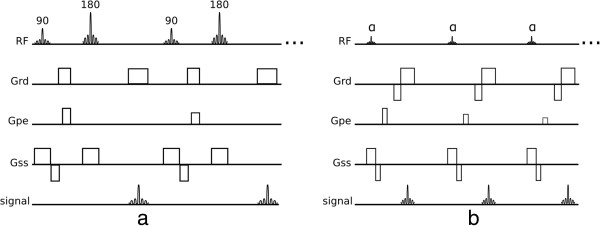
**Spin-echo and gradient-echo. a**) Conventional spin-echo pulse sequence. **b**) Conventional gradient-echo pulse sequence. There are 5 different events shown: *RF* - radio-frequency pulses, *Grd* - readout gradient axis, *Gpe* - phase-encode gradient axis, *Gss* - slice-selection gradient axis, and *signal* - acquired echoes.

Both spin-echo and gradient-echo pulse sequences play a major role in CMR. Spin-echo sequences refocus the excited signal with a 180° pulse or pulses (Figure 1a) which makes them robust to off-resonance effects and able to use longer TE (Time of Echo) values than gradient-echo sequences (Figure 1b). Off-resonance refers to a small deviation in the local spins' resonant frequency in relation to the nominated scanner centre frequency. This can be caused, for example, by main field inhomogeneities or magnetic susceptibilities.

### Spin-echo

Generally spin-echo sequences offer a greater flexibility in obtaining different contrasts (T1-weighted, T2-weighted, or proton-density weighted) depending on the choice of TE and TR (Time of Repetition). The main disadvantages of spin-echo sequences are their limited temporal-resolution and sensitivity to motion and flow.

**Figure 2:**
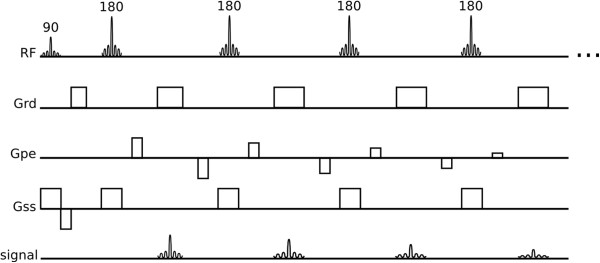
**Turbo spin-echo.** Turbo spin-echo or Fast spin-echo sequence.

Spin-echo acquisition times can be shortened by use of a multi-echo approach where multiple refocused echoes are acquired preceded by a single 90° excitation pulse (Figure 2) and this is known as turbo spin-echo (TSE), fast spin-echo (FSE) or rapid acquisition with relaxation enhancement (RARE). The reduced acquisition time permits the acquisition of a whole image within a breath-hold, reducing the chances of breathing motion while acquiring data. TSE has also been adapted to allow complete image acquisition within one heart-cycle known as HASTE. This approach increases the temporal-resolution at the expense of spatial-resolution and also uses a technique called partial-Fourier acquisition, which accelerates image acquisition time, as explained in the Acceleration Methods section later.

### Gradient-echo

**Figure 3:**
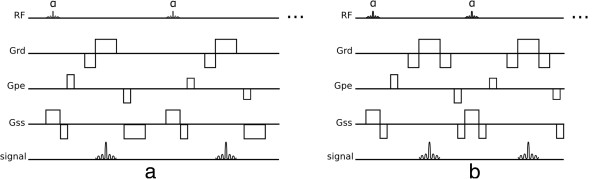
**GRE and bSSFP. a**) Spoiled gradient-recalled echo sequence. **b**) Balanced steady-state free precession sequence.

Gradient-echo sequences do not have a refocusing pulse; off-resonance effects are therefore not refocused making this class of sequences generally T2* weighted instead of T2, thus the need for shorter TE than spin-echo sequences because of the faster signal decay. Gradient-echo sequences generally provide faster image acquisitions than spin-echo sequences, and are thus very popular in CMR due to the demand for fast imaging. This rapid acquisition does not allow either the longitudinal or transverse magnetisation to fully relax between successive radiofrequency (RF) pulses, instead the magnetisation settles into a steady dynamic equilibrium (known as *steady-state*) during the multiple shots (excitations) of the sequence. Two strategies are commonly employed to deal with the remaining transverse magnetisation at the end of each TR: the remaining transverse magnetisation can be spoiled in Gradient-Recalled-Echo (GRE) sequence (Figure 3a), or it can be gradient refocused and reused as in the balanced-SSFP sequence (bSSFP) (Figure 3b). For the GRE sequence RF spoiling is often used to further reduce the impact of the remaining transverse magnetisation [6].

**Figure 4:**
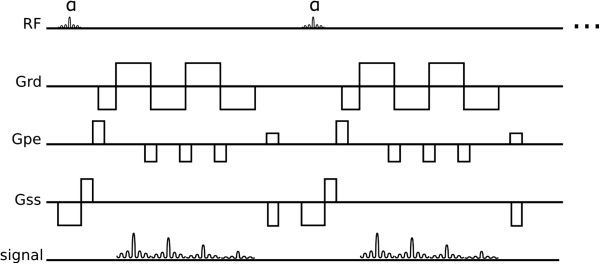
**Hybrid EPI.** Hybrid-EPI with 4 echoes per-shot.

The excitation pulses of GRE sequences are limited to a low flip-angle, and have inherently low SNR (Signal to Noise Ratio) but are less sensitive to off-resonance effects than bSSFP sequences. Both sequences can be repeated rapidly enough to acquire many echoes within a reasonable temporal period such that multiple cine frames can be acquired with a reasonable spatial-resolution within a breath-hold period. Alternatively, in a similar fashion to TSE, GRE sequences can be modified to acquire multiple echoes per excitation in a technique known as echo-planar-imaging (EPI). GRE sequences with an EPI readout can acquire a few echoes per excitation known as hybrid-EPI (h-EPI) or segmented-EPI (Figure 4); or acquire the whole data in just one excitation known as single-shot EPI. Due to quick signal decay in GRE sequences, single-shot EPI is seldom used. Please note that even though the echo formation during an EPI readout is of a gradient-recalled echo type, this technique can be used under a spin-echo signal envelope, i.e. by having an excitation and refocusing pulse prior to the EPI readout.

In a bSSFP sequence the transverse magnetisation is not spoiled but refocused by gradients, contributing to the next echo. The word *balanced* in balanced-SSFP means that the net area in each gradient axis is null within a TR (Figure 3b). bSSFP sequences can use higher flip-angles and shorter TRs than GRE, increasing SNR and decreasing acquisition time but at a cost of an increase in sensitivity to field inhomogeneity and frequency-offsets.

### Preparation pulses

Many different cardiovascular imaging sequences are combined with preparation pulses. These pre-pulses precede the host-sequence and can be used to suppress specific tissues such as blood or fat, enhance contrast weighting and add tags to the myocardium for example. Some of the most common preparation pulses are now described.

### Inversion pulses

**Figure 5:**
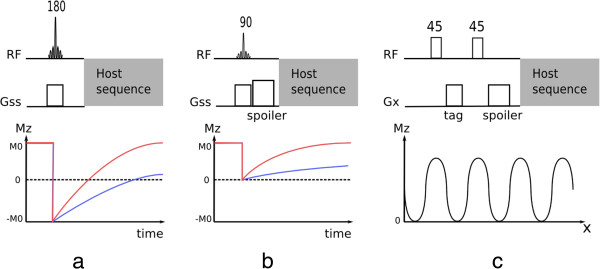
**Preparation pulses. a**) Inversion recovery pulse. **b**) Saturation recovery pulse. Both figures ***a*** and ***b*** show the longitudinal magnetisation recovery after the preparation pulse for two different T1 values (T1 red < T1 blue). Please note that in both ***a*** and ***b*** diagrams the effects on the longitudinal magnetisation by the host sequence are not considered. **c**) SPAMM tagging preparation showing the spatial modulation of the available longitudinal magnetisation following two 45 degree pulses prior to the host sequence.

Inversion pulses invert the longitudinal magnetisation from its resting (“equilibrium” or full-recovery) value usually labelled *M*_*z*_ = *M*_0_ to *M*_*z*_ = − *M*_0_ leading to signed longitudinal magnetisation recovery that is initially negative and then positive on its return to equilibrium M_0_ (Figure 5a). This type of pulse is often used to selectively null signal from tissues with a particular T1. Inversion pulses can be non-selective, inverting all longitudinal magnetisation over a large volume; spatially-selective by applying an amplitude modulation and a gradient; or spectrally selective (this type of inversion pulses can be used for example to invert or null the signal of fat only).

### Saturation pulses

Similar to inversion pulses, saturation pulses can also be used to modify contrast in an image (Figure 5b). Instead of inverting the magnetisation, a saturation pulse rotates the magnetisation into the transverse x-y plane where it is spoiled by a gradient, resulting in zero net magnetisation.

Following both inversion and saturation pulses, the longitudinal magnetisation starts to recover towards equilibrium, and image acquisition with one of the host sequences occurs during this recovery. These techniques are therefore called inversion-recovery or saturation-recovery preparation pulses. Different tissues have different T1 values and therefore different available signal during recovery; these recovery techniques allow image contrast between different tissues to be manipulated. It should be noted however that the final contrast is not just dependent on the preparation pulses but also on the host sequence.

Optimal saturation recovery preparation is generally achieved by the use of special RF pulse designs (composite [7], B_1_ Independent Rotation (BIR) [8]). Contrast manipulation is somewhat more limited than with inversion recovery pulses, although there are shorter recovery times from saturation. A major advantage of saturation recovery is that it does not require near full longitudinal recovery before the next saturation is applied, unlike inversion recovery, because the magnetisation is always reset to zero by the saturation pulse; therefore offering insensitivity to arrhythmias. These pulses are commonly used in first-pass perfusion imaging to enhance the contrast agent signal.

Saturation pulses can be also used to suppress unwanted tissues. This suppression can be either spatially-selective or spectrally-selective or both, reducing the signal from defined spatial regions in the body, or from defined nuclei with specific chemical shifts respectively. Saturation bands can be used for example to suppress aliasing of tissue outside the field of view or signal ghosting from flowing blood, while spectrally-selective saturation pulses are commonly used to null the signal of fat, commonly known as fat-saturation pulses. These types of saturation pulses differ from a saturation recovery preparation in the sense that the main objective is to null signal instead of manipulating contrast with spin-relaxation; thus this type of preparation is commonly followed by a spoiler-gradient in order to destroy any transverse magnetisation, and followed immediately by the excitation pulse in the host sequence, with no recovery time.

### Tagging pulses

Another form of preparation pulse used in CMR is the tagging pulse. This pulse introduces spatial tags, i.e. spatially periodic signal intensity modulation in the image and is commonly used to evaluate tissue deformation, such as myocardial strain throughout the cardiac cycle. Tagging pulses are commonly applied immediately after each R-wave followed by a segmented-image acquisition of multiple frames during the heart-cycle, to enable reconstruction of a *cine*. In a popular tagging preparation technique known as SPAMM [9], non-selective pulses are interleaved with a tagging gradient and followed by a spoiler gradient (Figure 5c). This preparation introduces a sinusoidal modulation in Mz along the tag gradient direction, represented by Gx in the Figure. This technique is usually applied separately in both in-plane spatial encoding directions and then combined in order to create a perpendicular grid. The contrast of the tag lines can be improved by summing two sets of images with complementary tagging pulses, in a technique called CSPAMM [10].

### k-space sampling

The data collected by the scanner needs to be converted to the final image. The acquired raw-data domain is commonly known as *k-space* and is mathematically related to the final image by a Fourier transformation. Thus each data value in k-space contains information about potentially any pixel in the final image, although a basic relationship can be considered between k-space and the image-space. The central region of k-space contains information about low spatial frequencies, i.e. mainly the contrast in the image, while the outer regions of k-space contain information about the high spatial frequencies, i.e. object-boundaries and edges [4]. This relationship between k-space and image-space allows the manipulation of artefacts and image contrast by modifying the way k-space is sampled. Many different k-space sampling trajectories have been proposed and used for cardiac imaging. In this section a brief introduction is made to some of these techniques, while associated artefacts are presented later on. Most of these techniques are simply a method of sampling k-space and therefore can be used with different sequences and techniques.

**Figure 6:**
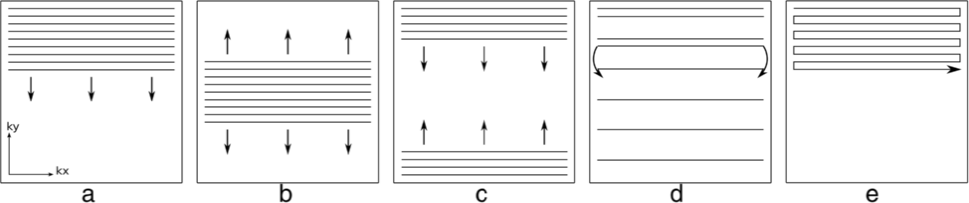
**K-space sampling phase order. a**) Sequential order. **b**) Centric order. **c**) Reverse centric order. **d**) Linear interleaved order. **e**) EPI trajectory (please note, no sampling is made during phase-encode (vertical) displacements).

The most common k-space sampling trajectories are Cartesian, i.e. each data point is located in a Cartesian grid, with each echo filling an entire phase-encoded line. This is by far the most simple and robust method, but not necessarily the most efficient. Cartesian k-space sampling can still be divided into different orders of acquiring the phase-encoded lines known as *phase-order* (also called view-order, or k-space order), different phase-orders present advantages and drawbacks in terms of artefacts as discussed later. The most common phase-orders are: sequential order; centric order; and reverse centric order (Figure 6a-c). For so called segmented acquisitions the sequential lines are either acquired more sparely and interleaving is used to fill the spare regions (Figure 6d); or simply fill k-space in sequential blocks over the raw-data.

**Figure 7:**
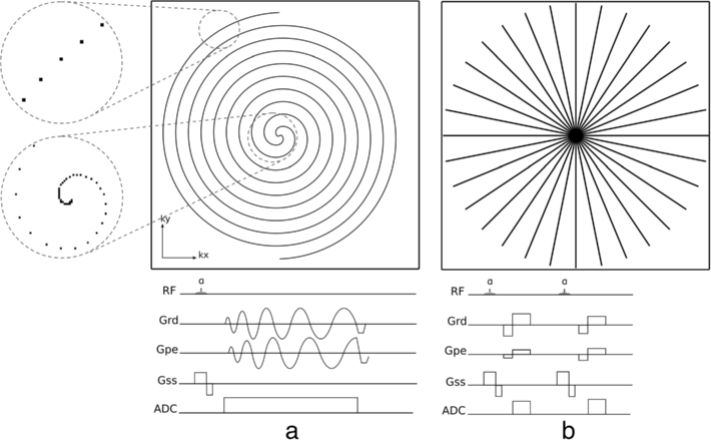
**Non-Cartesian sampling: Spiral and Radial. a**) Spiral k-space trajectory with two interleaves, the centre and edge of one interleave is zoomed in on the left. Below is shown a sequence diagram of the acquisition of one spiral interleave. **b**) Radial trajectory. Below is shown a sequence diagram for the acquisition of two radial lines.

Still in the realm of Cartesian trajectories we have EPI readouts, where more than one line is acquired in a gradient-echo train (Figure 6e). EPI uses bipolar readout gradients (Figure 4), which continuously produce echoes, making it a fast k-space sampling method. In its most common form EPI, although being Cartesian requires extra image processing steps due to half the echoes (odd echoes) being acquired in k-space with the opposite direction to even echoes. Although highly efficient, EPI is also very sensitive to many different artefacts, especially in its single-shot form and for this reason hybrid-epi is popular where shorter EPI trains are commonly acquired with only a few echoes or lines, instead of the whole of k-space.

k-space sampling can also have non-cartesian trajectories, with the most common being *spiral* or *radial*. In a spiral acquisition each k-space line follows a spiral trajectory (Figure 7a), this is efficient in the sense that it achieves a greater coverage of k-space with fewer shots than Cartesian. Also a spiral acquisition collects a circular area of k-space and therefore ignores the corners of k-space which are collected with a Cartesian acquisition but don’t necessarily contribute to the final image. Similarly to EPI, spiral acquisitions can sample the entire k-space either with one shot or with multiple spiral interleaves. The single shot approach is again more efficient but prone to artefacts. Spirals do not have a phase and frequency-encode gradient, instead there is a 2D readout gradient waveform that is sinusoidal with frequency and with varying amplitudes. The rate of increasing radius of each spiral line and also the rate of sampling in a line are defined by the Nyquist sampling requirements, which state that in order to reconstruct a signal from a sequence of samples, the frequency of samples must be at least double the maximum frequency in the signal and that if higher frequencies are present in the reconstructed signal then these will be misinterpreted or aliased as lower frequencies. These requirements are used to define the gradient waveform, although due to hardware limitations the gradients cannot reach their maximum amplitudes immediately from the beginning, and instead the amplitude is ramped up during the first few cycles. The result of this is an oversampling of data in the centre of k-space, with a higher density of data points than in the edges (Figure 7a).

Radial sampling was the first k-space sampling trajectory to be used in MRI with a backprojection image reconstruction [11], analogous to Computed Tomography. In a radial trajectory the k-space is sampled with radial spokes that pass through the centre of k-space (Figure 7b). Image reconstruction in radial sampling can either be with a back projection algorithm or, most commonly, gridded into a Cartesian matrix which is then reconstructed as Cartesian data. One potential advantage of a radial acquisition is the shorter minimum TE as there is no phase-encoding required. To satisfy the Nyquist sampling requirements, the number of acquired radial k-space lines must be greater than with Cartesian sampling by a factor of π/2; although occasionally mild undersampling may not compromise the diagnostic quality of the image, such as in contrast-enhanced vascular imaging. Variations of radial acquisition exist such as Linogram and PROPELLER. The explanation of these methods falls outside the scope of this article, but the interested reader is referred to the respective bibliographical references [12,13].

Similar to Cartesian trajectories, non-Cartesian trajectories can also be modified in order to change image contrast by changing TE and for example T2* sensitivity. In both radial and spiral acquisitions, sampling can start either at the centre of k-space or at the edge, resulting in a short or longer TE respectively.

Non-Cartesian methods suffer a definite disadvantage in their sensitivity to even a few microseconds of synchronisation errors among the gradient waveform axes and also of these with data sampling. Cartesian scanning (except EPI) is much more tolerant of such errors.

### Acceleration methods

**Figure 8:**
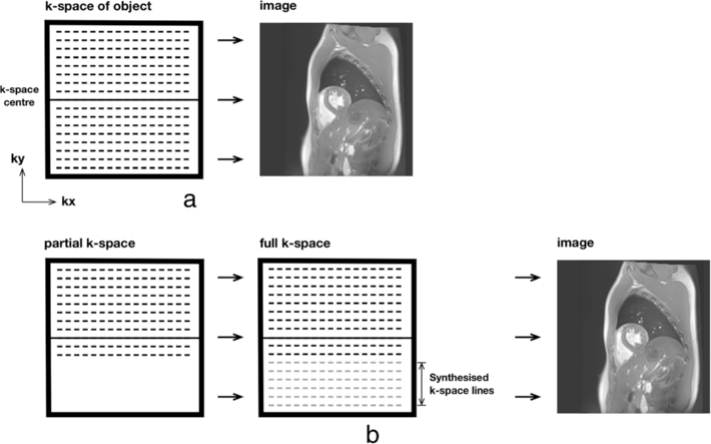
**Partial-Fourier. a**) Full k-space sampling reconstruction. **b**) Partial-Fourier reconstruction. Prior to Fourier-transform into the image-space, the missing k-space lines are synthesised from acquired lines based on conjugate symmetry.

Image acquisition in cardiovascular imaging is often accelerated with image reconstruction techniques that share the basis of only acquiring part of the whole k-space, such as *parallel imaging*, and *partial-Fourier*. In the case of partial-Fourier, one half of k-space is only acquired partially (Figure 8); the missing k-space samples are either considered to be of zero amplitude (known as zero-filling) or by taking into account the fact that one half of k-space should be the complex conjugate (k-space complex numbers where the imaginary components have the opposite sign) of the other half so that the missing points can be calculated and the k-space data reconstructed using a so called homodyne reconstruction [14]. Partial-Fourier reduces SNR and reconstruction is usually poor in regions of rapid phase changes due to magnetic susceptibility.

Parallel imaging is another method of accelerating image acquisition without sacrificing spatial-resolution [15]. In parallel imaging the spacing in k-space between acquired phase-encode lines is bigger than that required by the Nyquist sampling requirement for the phase-encoded FOV. This increase in line spacing is given by the acceleration factor *R*, for example an *R*=3 means that only one in every three lines is acquired. The result is a FOV reduced by a factor of 3, and aliasing or wrapping of the imaged object into the opposite edge of the image, if bigger than the reduced FOV. Parallel imaging requires phased-array coils, since it uses the coils’ individual spatial sensitivities (information about the spatial signal response profile of each coil) to either unwrap the object after image reconstruction or fill the missing k-space lines before the image reconstruction. Several parallel imaging methods are available with the most common being GRAPPA and SENSE [16,17].

**Figure 9:**
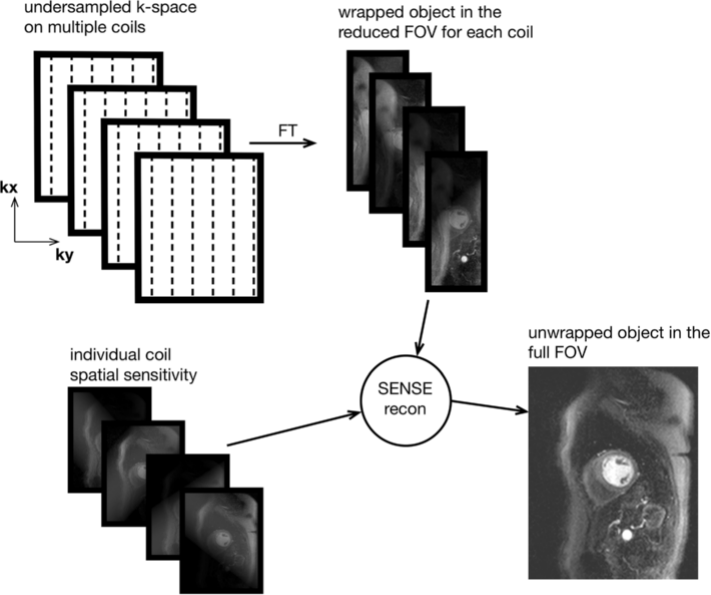
**SENSE reconstruction.** Diagram depicting parallel imaging reconstruction with SENSE. The final image is calculated by using the coil wrapped images together with their sensitivity maps.

In SENSE the incomplete k-space data is first converted into the image-space and then unwrapped using the multiple coil information (Figure 9), while with GRAPPA the missing k-space lines are generated for each coil using the acquired data of all the coils, i.e. undersampling effects are addressed before Fourier transformation into the image-space. The coils’ spatial sensitivities required by these methods can be acquired with low spatial resolution during image acquisition either by fully sampling the centre of k-space, while keeping the outer regions undersampled, or by acquiring a pre-scan. Parallel imaging reduces the overall SNR of the image because of the reduced number of acquired phase-encode lines, but it also adds an additional spatially varying SNR penalty that is dependent on the positions and distribution of the coils in the array along the phase-encode direction. The better the positioning and distribution of the coils the lower the SNR penalty.

Either SENSE or GRAPPA can also be combined with another form of temporal undersampling (temporal filtering technique) known as UNFOLD [18], these are then usually known as TSENSE [19] and TGRAPPA [20] respectively. Combining UNFOLD with parallel imaging techniques improves coil sensitivity profile estimation.

**Figure 10:**
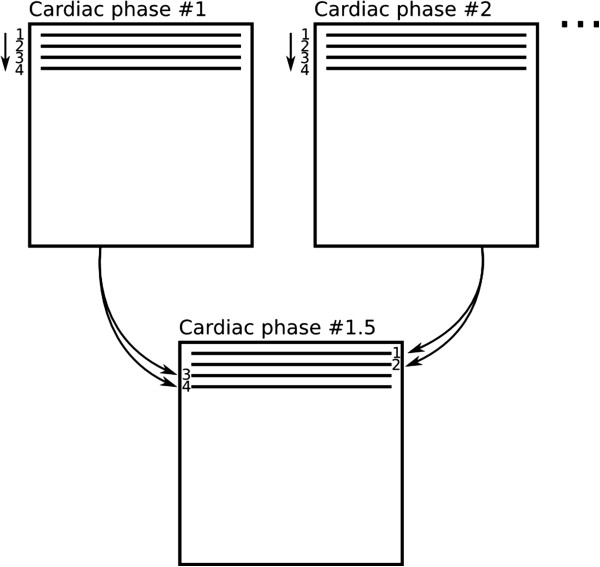
**View-sharing.** An intermediate cardiac-phase (**bottom**) is constructed from the acquired data of the first and second cardiac-phases (**top**). In this diagram only one k-space segment with 4 phase-encode lines is shown, this process is repeated for all subsequent segments.

View-sharing is another image reconstruction technique, commonly used to increase the temporal-resolution by reconstructing intermediate frames (also called *cardiac phases*) with the raw-data of the surrounding frames in cine acquisitions [21,22] The data of the intermediate cardiac-phases is not interpolated, but simply assembled from the existing data (Figure 10).

### Cardiac imaging

In this section we describe the most common cardiovascular imaging applications to provide a context to the following section on artefacts.

### Morphology

For cardiovascular morphology, a double-inversion RF pulse preparation is commonly added to a turbo spin-echo sequence, removing the blood signal and providing a good contrast between the myocardium and blood, and is commonly referred as *black* or *dark-blood* preparation [23]. Image acquisition can be achieved within a breath-hold, yielding a reasonably high spatial-resolution, or faster within a heart-cycle, with a reduced number of phase encoding steps and partial-Fourier, using a technique known as HASTE (Half-Fourier Acquisition Single-shot Turbo Spin-echo) [24].

**Figure 11:**
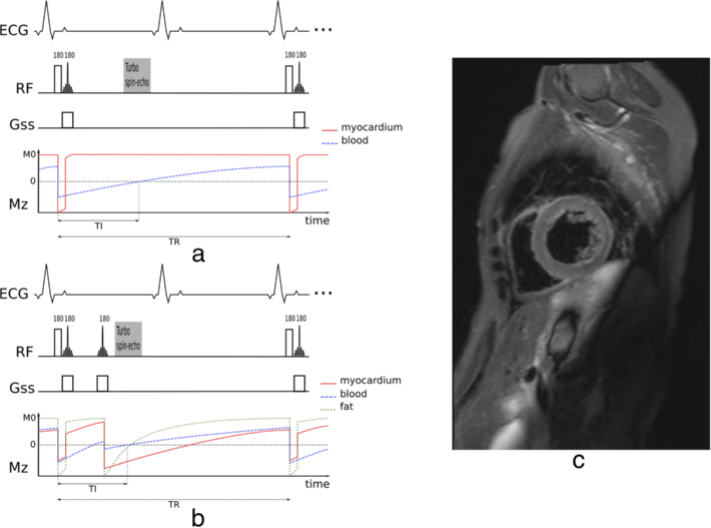
**Black-blood imaging. a**) Double-inversion preparation (the red line represents the myocardial longitudinal magnetisation (Mz) in the image slice, the dashed blue line represents the inverted blood Mz including that that has flown into the image-slice at the time of image acquisition, and the TI represents the null point of blood). **b**) Triple-inversion preparation or as it is often called STIR preparation (the red line and the dashed blue line represent the myocardial and the flowing blood Mz as in the previous figure, the dotted green line represents the fat Mz in the image slice). Please note that in both *a* and *b* diagrams, the effects of the host sequence are not considered for the Mz curves. **c**) Example of a short-axis STIR TSE image.

The double-inversion preparation consists of two inversion pulses applied after the R-wave. The first inversion pulse is spatially non-selective inverting the magnetisation in the whole imaging volume while the second inversion pulse selectively re-inverts the magnetisation in the image slice to be acquired (Figure 11a). The magnetisation in the image slice is therefore restored to its original state while the magnetisation outside the image slice slowly recovers from its inverted state. Image excitation and readout is at the time when the originally inverted blood magnetisation outside the image slice recovers to zero and cannot produce a signal or is nulled. Depending on the heart-rate this normally occurs during mid-to-late diastole after an inversion time of 400-600 ms. While waiting for this magnetisation nulling, due to blood flow, the non-inverted blood in the image slice is replaced by the originally inverted blood during systole and early diastole, resulting in a dark-blood image. Image acquisition in mid-to-late diastole not only avoids rapid cardiac motion but it also allows the heart to go back to approximately the same position in the image slice as when the double-inversion pulses were applied earlier in the cycle. This technique is only suitable for single-slice and normally diastolic imaging, but it is compatible with a wide range of different heart-rates, since faster heart-rates require smaller inversion times in the preparation, and therefore the magnetisation preparation can still be accommodated in the shorter RR interval.

The double inversion preparation can be modified to additionally suppress the signal of fat. This is accomplished by adding a second spatially selective inversion pulse closer to the host turbo spin-echo sequence (Figure 11b), known as triple-inversion recovery or short-tau inversion-recovery (STIR). This additional pulse is applied when the magnetisation of the flowing inverted blood is slightly positive, in this way the signal of the flowing blood and fat are close to their null point at the time of imaging. Because of the third inversion, myocardium is imaged while it is at negative magnetisation, but the magnitude image discards the polarity, resulting in a bright myocardial signal.

### Global function

**Figure 12:**
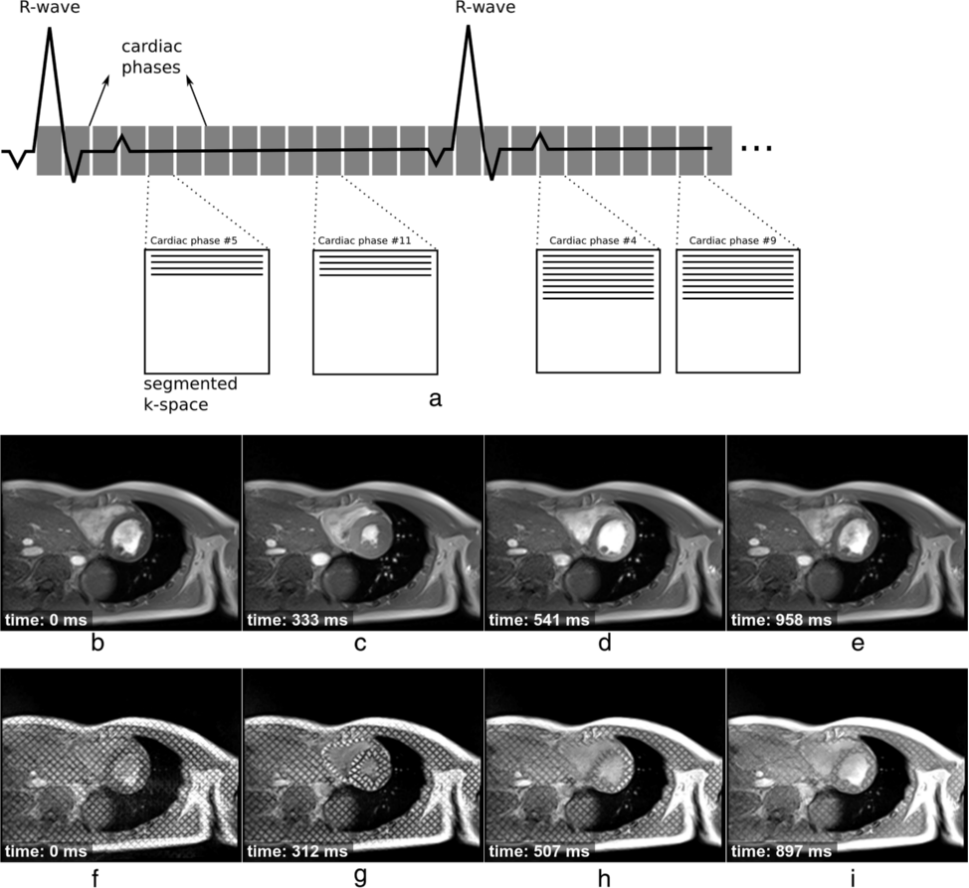
**Cine acquisition. a**) Cine acquisition diagram showing 15 cardiac phases (cine frames). For each heart cycle in this example a segment with four phase-encode lines is acquired for each cine frame. K-space is being filled in a sequential manner. **b**-**e**) 4 frames out of 25 of a bSSFP short-axis cine showing the acquisition time after the R wave. **f**-**i**) 4 frames out of 25 of a GRE short-axis cine where a tagging pulse was applied immediately after each R-wave. The time of acquisition after the R wave is also shown.

bSSFP and GRE sequences are commonly used to assess cardiac function [25-28]. These sequences are acquired and viewed in cine mode, allowing the visualisation of cardiac motion and morphology throughout the heart-cycle. The acquired data can also be used to measure stroke volume, ejection fraction and wall thickness. To build high spatial and temporal-resolution frames (known as cardiac phases), image acquisition needs to be made over multiple cardiac cycles, known as segmented acquisition. For each heart-cycle only a number of lines of data per segment (known as k-space segment) are acquired for each cardiac-phase (Figure 12). Several heart-cycles (normally within a breath-hold) are therefore needed to fill out the entire k-space of each cardiac-phase.

Good ECG gating or triggering is important to obtain good quality images, and different approaches exist. Cine acquisitions can be prospectively triggered or retrospectively-gated [29,30]. For prospective triggering, each R-wave triggers the acquisition of a new k-space segment for each cardiac-phase. There are two forms of retrospectively-gated cine imaging. For the first a k-space segment is repeatedly acquired over a predefined time window (bigger than the maximum R-R interval), during the subsequent time window the next segment is acquired. The k-space segments acquired together are labelled with their time during the heart-cycle which is used during image reconstruction. This form of retrospective-gating takes longer to acquire than the second form where the QRS complex is detected and used to instantly advance to the next segment on-the-fly. These retrospective approaches can potentially be less susceptible to small heart-rate variations and they also allow the acquisition of cardiac-phases during late-diastole. Irrespective of the form of gating the temporal-resolution of cines is usually improved with view-sharing (Figure 10).

Cardiac function can also be complemented, to measure regional function, with a tagging sequence (see Section on Tagging Pulses, Figure 5c, and bottom of Figure 12). Tagging data is acquired in the same way, i.e. segmented cine, but with extra tagging pulses immediately after each R-wave trigger and before the cine sequence. The acquired tagging data can be analysed to compute several cardiac parameters including myocardial strain and torsion.

### Blood flow

**Figure 13:**
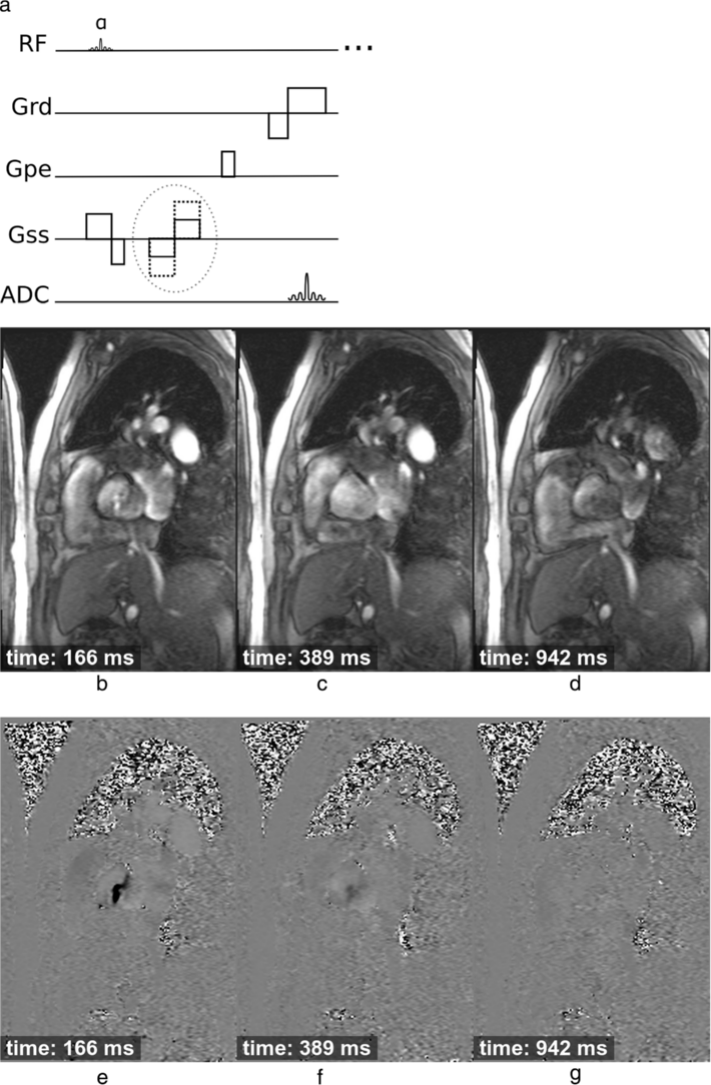
**Blood flow imaging. a**) GRE sequence which is repeated with two different bipolar gradient moments (dashed circle). **b**-**g**) Cine frames showing through-plane blood flow of the aortic valve at three different time points of the heart cycle in a patient with valve stenosis: **b**-**d**) 3 of 20 magnitude cine frames showing the acquisition time after the R wave, **e**-**g**) the respective phase velocity frames.

Gradient-echo sequences can also be used to measure blood flow velocity using a technique known as phase contrast velocity mapping [31-34]. Bipolar gradient waveforms that are inherently part of gradient-echo sequences, naturally introduce velocity related phase shifts to the signals that are reconstructed into the image. By acquiring two gradient-echo images with different but well defined gradient waveforms it is possible to produce a well defined velocity related phase difference between the two reconstructed images. By reconstructing a phase difference image a velocity map is produced, which shows velocities in the direction of the different gradient waveforms of the two acquired sequences (Figure 13).

The phase differences can be well defined and velocity-encoded in any or all three perpendicular spatial directions, but are usually applied to just one axis at a time, giving an estimate of the blood flow velocity in each voxel through the chosen direction. It is common to measure the blood velocity in a cross section of a particular vessel. Data is usually acquired using a segmented approach, as described earlier, over a breath-hold. A cine of phase difference maps is reconstructed showing the temporal changes of the velocity throughout the cardiac cycle. Other sources of phase differences, due to field inhomogeneities for example, are the same on the two sets of phase images and these are removed when the images are phase subtracted.

### First-pass myocardial perfusion

**Figure 14:**
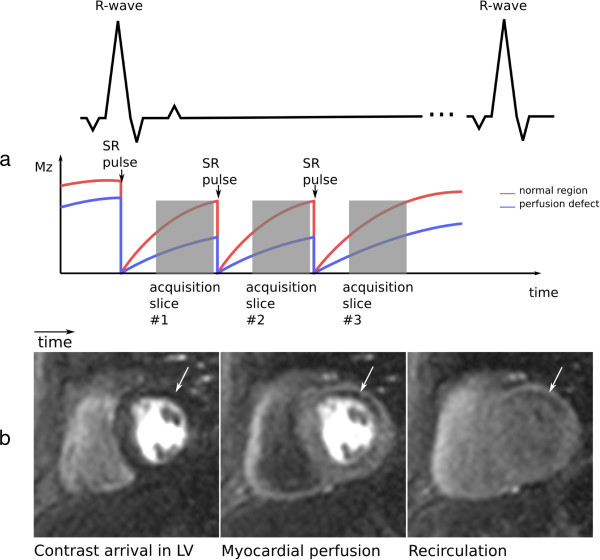
**Myocardial first-pass perfusion. a**) Multi-slice first-pass myocardial perfusion imaging diagram. The red and blue lines represent the longitudinal magnetisation available in a normal region and in a hypoperfused region of the myocardium respectively. SR - saturation recovery preparation. **b**) Basal short-axis slice showing Gd arrival at three different times, from left to right: Gd arrival in the LV, myocardial perfusion of Gd, and after Gd first-pass. The arrow points to an hypoperfused region.

Myocardial first-pass perfusion imaging has the requirement that the temporal and spatial-resolution must enable a good myocardial coverage for each heart beat in order to accurately image the first-pass of a Gadolinium (Gd) based contrast agent [35]. One of the fast sequences: GRE, h-EPI, or bSSFP are invariably used as these sequences permit the acquisition of a full image in a fraction of the heart-cycle and allow several magnetisation prepared image slices to be acquired (Figure 14).

Perfusion images are commonly T1-weighted by a saturation-recovery preparation pulse that zeroes the entire longitudinal magnetisation a defined interval prior to the image acquisition. Image acquisition occurs during signal recovery from saturation, allowing a good visualisation of the Gd distribution due to its T1-shortening effect: myocardial regions with perfusion defects will have less Gd and are therefore darker than normal regions.

GRE and bSSFP sequences are commonly acquired with a sequential k-space phase-order, while h-EPI generally uses a perfusion-tailored centric interleaved phase-order described by Ding et al [36]. This interleaved acquisition order minimises the TE in the central lines (effective TE) while providing a good T1-weighting for superior contrast enhancement, but it can lead to artefacts as described later.

### Viability

Myocardial viability is commonly assessed with a technique known as Late Gadolinium Enhancement (LGE). A segmented GRE sequence with an inversion pulse preparation is normally used [37]. The acquisition is ECG gated to diastole for every other heartbeat, as for best results at least a beat needs to be skipped in order to allow T1-recovery prior to the next inversion pulse. One image is typically acquired during each breath-hold, and 10 to 12 breath-holds are needed to cover the entire heart (stack of short-axis slices). The slices are sometimes repeated with swapped phase-encode direction, partly to guard against artefacts as described later, but often also for multiple studies during washout.

**Figure 15:**
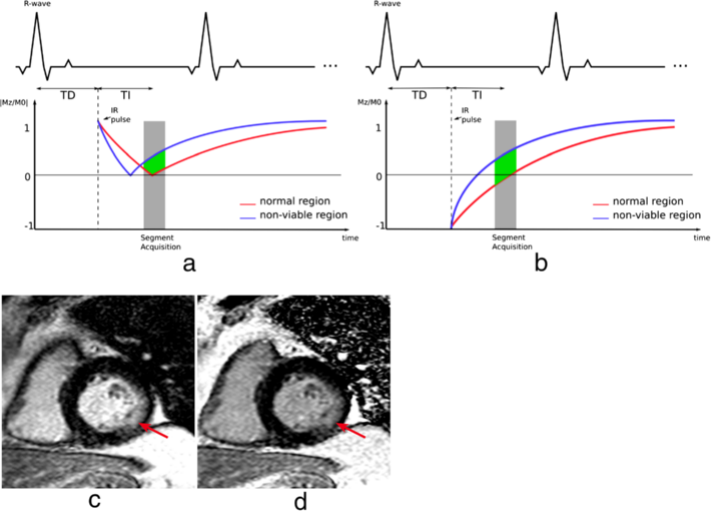
**Late gadolinium enhancement. a**) Diagram showing the magnitude reconstruction signal following an inversion pulse designed for LGE. The red and blue lines represent the available longitudinal magnetisation in the normal tissue (red) and the non-viable tissue (blue). **b**) Diagram of phase-sensitive reconstruction. In both diagrams ***a*** and ***b***, the difference in signal during image acquisition between both tissues is shown in green. TI - time of inversion, TD - trigger delay, IR - inversion recovery preparation. Note that for some manufacturers TD is measured to the centre of k-space. **c**-**d**) Example of magnitude and phase-sensitive reconstruction respectively of a short-axis image with myocardial LGE in the lateral wall (arrows) and good nulling of normal myocardial signal.

The TD (trigger delay) and TI (time of inversion) are chosen to acquire data at diastole and with null signal from viable myocardium (Figure 15a). Relatively bright signal is visible in non-viable regions of the myocardium, where Gd tends to accumulate, subsequently shortening the T1 in that region and providing high contrast against the nulled viable regions. In cases where the operator lacks experience, choosing the right TI might be challenging; a technique known as phase sensitive inversion recovery (PSIR) [38] can be used to enable adjustment of contrast and nulling and to relax the dependence on the TI value (Figure 15b). Single-shot bSSFP sequences can be used to accelerate imaging for patients with arrhythmia or those unable to breath-hold with conventional protocols, but these have the cost of lower spatial-resolution [39].

### Coronary angiography

**Figure 16:**
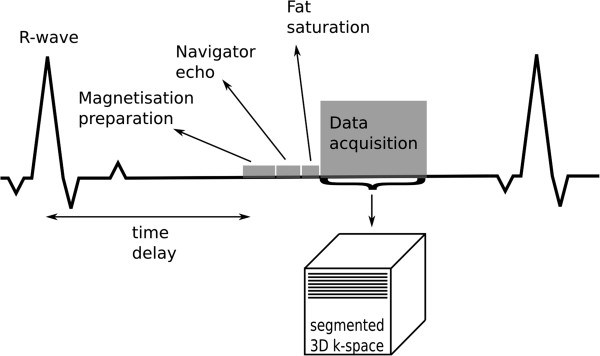
**Coronary angiography.** Triggered segmented 3D angiography acquisition diagram. The time delay is used to acquire the data at mid-diastole. The magnetisation preparation can be used to enhance the myocardial-to-blood signal contrast. The navigator echo is used for respiratory correction and control, followed by a fat saturation pulse. Several phase-encode lines are then acquired during the data acquisition window within each heart-cycle.

A segmented 3D ECG-triggered acquisition is typically used to image the coronary arteries (Figure 16) [40-42]. The most common sequence used is bSSFP, and data is collected during mid-diastole to minimise cardiac motion. A high spatial-resolution is required to image the coronaries, and a 3D acquisition is inherently lengthy. Respiratory motion is therefore an issue, and a navigator guided acquisition during free-breathing is typically applied. A navigator echo is a 1D image of a selected column of tissue normally orientated in the head foot direction through the dome of the diaphragm. The 1D image can be reconstructed in real-time at least once every cardiac cycle to monitor respiration through the position of the diaphragm edge. Reasonable volume coverage with high SNR can be achieved with free-breathing techniques.

Contrast-enhanced GRE is common at 3T, taking advantage of the increased blood signal at higher field strengths [43]. A T1-weighted (non-selective inversion recovery) GRE is used with a TI between 230 and 320 ms to suppress the myocardial signal during the slow infusion of contrast agent. Using a contrast agent increases the contrast between blood and the background signal, however the complexity and limitations of the study increase; contrast-agent usage is limited, and finding the optimum time between injection and imaging is a common difficulty.

Another imaging technique used for imaging the coronary vessel wall is a TSE dark-blood approach; in a similar way to morphology sequences, a dark blood double inversion pulse is used in combination with frequency selective fat suppression prior to image acquisition [44].

### Cardiovascular magnetic resonance artefacts

Following the description of CMR techniques, this section introduces the main purpose of this manuscript, the description of the most common and problematic artefacts in CMR. The causes of these include motion (respiratory, cardiac, and blood flow); Gibbs ringing; aliasing; chemical-shift; and B0-inhomogeneities. In the following sections these different artefact sources will be discussed with particular regard to their physical basis and implications for the different sequences and applications.

### Motion

The overall motion of the heart is a complex mixture of cardiac motion associated with its cyclic pumping and respiratory motion which results in an additional twisting and volumetric distortion. The respiratory motion is relatively unpredictable and can vary considerably from person to person and from time to time. Cardiac motion has been reasonably well controlled over the years by detecting the QRS complex of the ECG and triggering the acquisition at a certain delay following this. Obviously ECG triggering works best when there is low variation between beats; as discussed later, arrhythmias and ectopic hearts will cause artefacts.

The respiratory motion has in recent years been largely controlled by acquiring the data over the period of a breath-hold, although this can translate into a long acquisition window within the cardiac cycle, thus potentially including periods of more rapid cardiac motion. Restricting the acquisition to a period of mid diastole where the heart is reasonably still is sometimes not feasible during a breath-hold, especially for patients that have considerable problems in holding their breath longer than a few seconds. For patients with very rapid heart-rates it can also be difficult to find a “motion free” acquisition window. Respiratory gating is another technique that allows the removal of gross respiratory motion artefacts by restricting data acquisition to the expiratory pause, this enables longer scans with shorter acquisition windows within the cardiac cycle, which in turn reduces cardiac motion problems. Respiratory gating is most commonly used in 3D imaging, in particular in imaging of the coronary arteries due to the high spatial-resolution (larger data acquisition matrices) and coverage required. The imaging time makes breath-hold imaging impracticable.

A moving object will change both the phase and magnitude of its k-space components. Motion during image acquisition will therefore introduce artefacts, and these can be divided into two categories, motion during the acquisition of one phase-encode line *intra-view*, and motion between different phase-encode lines *inter-view*. For most sequences intra-view motion at typical myocardial and respiratory speeds can be ignored, although rapid blood flow in major vessels can be an issue. Inter-view motion artefacts can be caused by cardiac motion and or breathing motion and are very dependent on the nature of the motion in relation to the k-space coverage.

Artefacts can also be created by motion between different components of the sequence, for example between the timing of preparation pulses and image acquisition for a black-blood sequence. The next subsections describe the basics of motion artefacts introduced in cardiac studies by breathing motion, cardiac motion, and blood flow.

### Breathing motion

**Figure 17:**
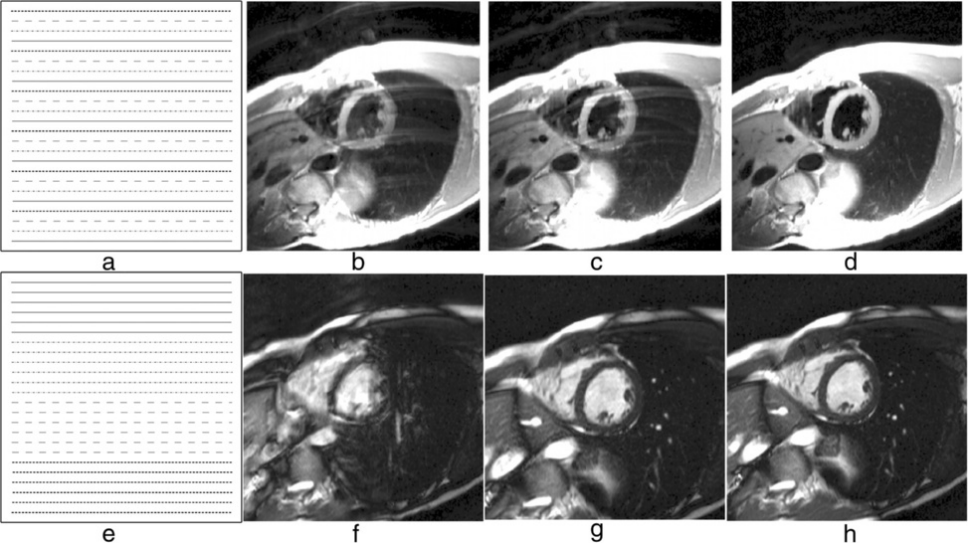
**Breathing motion artefacts with two different phase-orders.** This figure shows the artefacts caused by changes in respiratory position at different times during a breath-hold acquisition. **a**) Shows an interleaved segmented phase-order (as used in a black blood TSE sequence), with 4 segments, each with 6 phase-encode lines, each colour/line-type represents a different segment. **b**) The effects of a respiratory movement in the middle of the acquisition. **c**) The effects of a respiratory movement at the end of the acquisition. **d**) A good breath-hold. **e**) Shows a block sequential phase-order case (as used in a cine bSSFP sequence), with 4 segments, each with 6 phase-encode lines, each colour/line-type represents a different segment. **f**) The effects of a respiratory movement in the middle of the acquisition. **g**) The effects of a respiratory movement at the end of the acquisition. **h**) A good breath-hold. With a block sequential phase order the central region of k-space is acquired during a certain well defined period, and not spread throughout the whole acquisition window, therefore if no respiratory motion happens during this period, the artefacts are less conspicuous.

Most cardiac sequences are segmented, i.e. the acquisition of one image is divided into multiple heartbeats and the acquisition window in each heartbeat is restricted, in order to reduce cardiac motion artefacts and blurring. On the other hand, respiratory motion introduces k-space inconsistencies between different segments. Breathing artefacts will depend on the phase-encoding order used, and the timing of the motion. If, for example, motion only occurred when sampling the edges of k-space, then motion artefacts would result in blurring of the edges of the moving object in the phase encoding direction. If, on the other hand, the central regions of k-space were affected then this would result in a more significant ghosting and image degradation (Figure 17). In general if breathing motion is periodic during the acquisition of k-space in the phase encode direction, it results in a number of defined “ghost” artefacts distributed in that direction on the image. As can be seen from Figure 17, for acquisition sequences that employ an interleaved segmented coverage of k-space then a single movement or drift in the respiratory position will have a similar impact to periodic breathing motion. On the other hand for sequences that acquire k-space in a block sequential manner a single movement, as long as it doesn’t coincide with the centre of k-space, or similarly a drift in position, will cause some blurring but will generally cause less impact through ghosting. Different segmented sequences have different optimal phase-encoding orders, and therefore will be affected by respiratory motion differently. Generally, to avoid sudden signal amplitude and or phase discontinuities through k-space, which would lead to other artefacts, the Turbo-Spin-Echo and conventional gradient echo sequences acquire the data with an interleaved manner and the balanced SSFP sequences acquire in a block sequential manner. However, it should be noted that the exact methods may vary between manufacturers and even for the same manufacturer over time.

To avoid breathing motion artefact problems, the total imaging time is kept short, and suitable for a breath-hold. If the patient is unable to hold their breath, the total imaging time needs to be reduced. Possible solutions include the use of parallel imaging, although the reduction of SNR in some applications such as LGE imaging may prohibit this; or the reduction of overall k-space lines acquired, thus reducing phase-encode spatial-resolution. Another solution is to reduce the temporal-resolution, increasing the data lines per cardiac cycle and the imaging window of each cardiac-phase. This may have the cost of increasing cardiac motion problems, especially if imaging during rapid cardiac motion stages.

**Figure 18:**
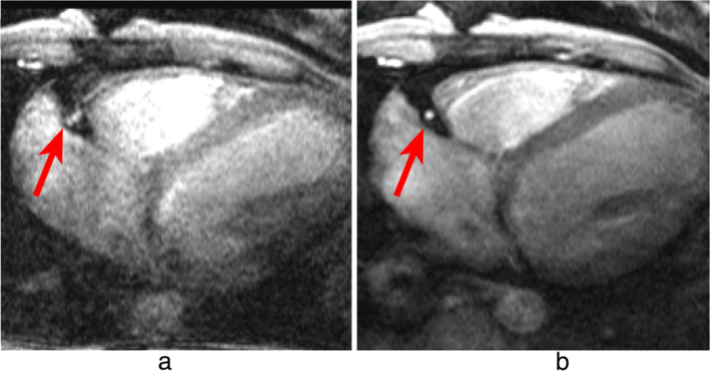
**Right coronary imaging with cardiac motion artefacts.** Magnitude images from a phase-velocity mapping study of the right coronary artery (arrows) acquired during systole. **a**) Breath-hold with long acquisition window during each cardiac-cycle, **b**) Retrospective respiratory gated with shorter acquisition window during each cardiac-cycle. Image *a* is considerably degraded due to cardiac motion in the longer acquisition window. (Adapted and reprinted, with permission, from reference [50]).

Breathing motion is one of the biggest challenges of MR coronary angiography. During cardiac motion, the coronaries have been shown to shift position from 5 to 20 mm [45], therefore the imaging window is limited to mid-diastole when the heart is relatively still. Often the cardiac timing is adjusted according to the coronary movement assessed from a long-axis cine. 3D coronary angiography imaging happens during free-breathing and uses navigator echo techniques to monitor respiratory motion (Figure 18). This information can be used simply to either accept/reject [46] the signal of that heart-beat or it can also be used to correct the slice position in real-time compensating for the breathing-motion of the coronaries [47] and increase the acceptance window allowing a reduction in the total imaging time. The most common navigator technique is to track respiratory motion with the dome of the right hemidiaphragm. The tracking factors that relate the diaphragm motion to the motion of the coronaries are highly variable from subject to subject [48,49] and therefore either an average is used compromising image quality, or specific tracking factors need to be measured for each patient at the cost of increasing study complexity. When correcting for motion of the coronary arteries, one possible problem that might arise is ghosting from nearby structures that do not move in a rigid way with the heart although this can potentially be reduced by saturation bands.

In general if imaging during a breath-hold, a saturation band can be applied positioned over the anterior chest wall, to suppress any motion-ghosting from this if the breath-hold is imperfect. This is a common technique in LGE scans.

### Cardiac motion

Cardiac motion is another source of inter-view motion artefacts. Cardiac motion is mainly a problem in sequences where the data acquisition window includes periods of rapid cardiac motion. Blurring can be caused by motion during acquisition of a long segment as illustrated for coronary motion in Figure 18a.

Another good example is first-pass myocardial perfusion imaging, where several images are fully acquired during each heartbeat; therefore image acquisition windows are long and spread across the whole of the cardiac cycle, including rapid cardiac motion stages. The heart will go through contraction and expansion as different phase-encode lines are acquired; motion happens both in-plane and through-plane, resulting in artefacts. Acquisition windows for one perfusion image are approximately 100 ms for GRE and bSSFP sequences and 70 ms for h-EPI, with parallel imaging with an acceleration factor of 2.

**Figure 19:**
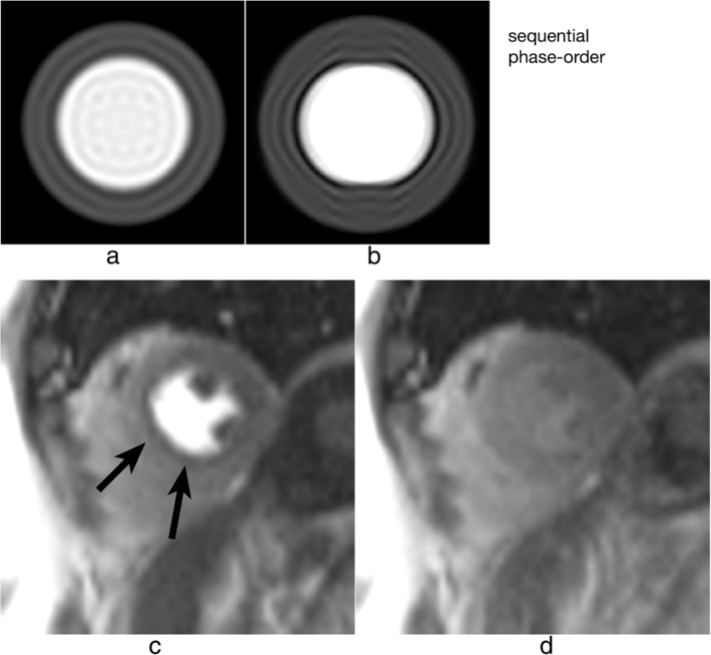
**Myocardial perfusion and cardiac motion artefacts (sequential phase-order). a**) Numerical simulations of a short-axis image (including Gibbs ringing) with no in-plane motion. **b**) Same as ***a*** but with in-plane cardiac motion (myocardial radial contraction) with a sequential phase-order acquisition. **c**) *In vivo* short-axis image of a perfusion scan with a bSSFP sequence with a sequential phase-order acquisition. A subendocardial dark rim artefact is visible likely to be a superposition of motion and Gibbs ringing artefacts (arrows). **d**) Same as ***c*** but after first-pass; the contrast between the LV and myocardial signal is reduced and the dark rim artefact is no longer visible.

For a Cartesian sequential phase-order, a continuous motion results in banding artefacts next to sharp edges [51], which looks similar to Gibbs artefacts (described later). These motion artefacts are illustrated using a numerical simulation and an *in vivo* example in Figure 19 for first-pass myocardial perfusion. Therefore for GRE and bSSFP sequences that typically use this phase-order, motion ringing artefacts can be superimposed with Gibbs (see *Gibbs Ringing* section) and possibly mimic real subendocardial perfusion defects during first-pass. The motion ringing magnitude is dependent on the signal difference across the edges, motion artefacts are thus expected to be problematic during first-pass when there is a large contrast between the LV blood pool and the myocardium. The subendocardial dark rim artefacts created by motion ringing are typically darker than the ones created by Gibbs ringing [52].

**Figure 20:**
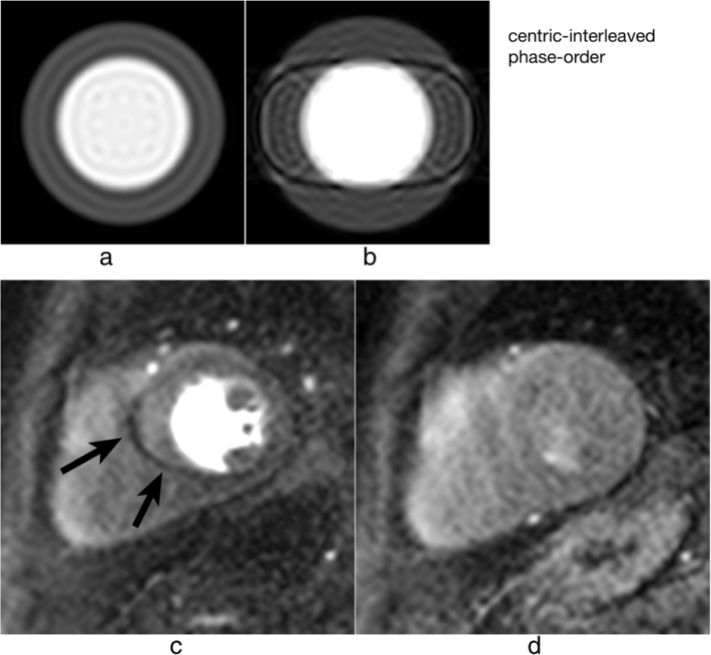
**Myocardial perfusion and cardiac motion artefacts (centric-interleaved phase-order). a**) Numerical simulations of a short-axis image (including Gibbs ringing) with no in-plane motion. **b**) Same as *a* but with in-plane cardiac motion (myocardial radial contraction) with a centric-interleaved phase-order acquisition. **c**) *In vivo* short-axis image of a perfusion scan with an h-EPI sequence with a centric-interleaved phase-order acquisition. A typical motion artefact is visible in the septal wall. This is no longer a subendocardial dark rim as shown in the previous Figure for a sequential phase-order, but ghosting from the endocardial border offset along the phase-encode direction (arrows), similar to the numerical simulation shown in ***b***. **d**) As in the previous Figure, motion artefacts are no longer visible after first-pass due to the reduction of signal contrast between the LV and myocardium.

As discussed earlier the h-EPI perfusion sequence is commonly used with a centric interleaved phase-order tailored for perfusion [36], minimising the effective TE. This sequence is the most robust to motion artefacts, not only because it is the fastest of the three most common perfusion sequences, but also because of its different phase-order; cardiac motion artefacts do not result in subendocardial dark rim artefacts, but in dark ghosting of the endocardial border along the phase-encode direction (Figure 20). The centric interleaved phase order is thus useful to differentiate cardiac motion artefacts but it also makes the h-EPI sequence very sensitive to frequency-offsets as described below.

In general, whatever the k-space acquisition scheme, in order to minimise cardiac motion artefacts it is important to keep the image acquisition time as short as possible in each heartbeat. Possible approaches, therefore, include using a fast EPI readout, and/or parallel imaging. Another solution is to aim imaging for timings of the heart-cycle where the heart is relatively still, although in some applications such as multi-slice myocardial perfusion imaging this is not possible for all slices, especially when patients are under stress with increased heart-rates. Also, for this specific application the slice-order could result in some slices being more motion affected than others. This slice-order, however, is not generally under the control of the scan operator.

**Figure 21:**
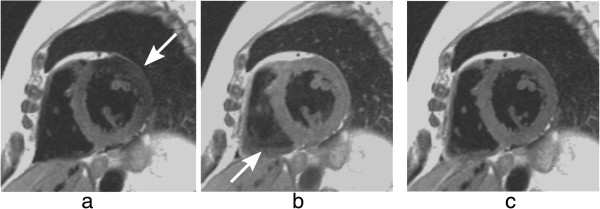
**Dark-blood imaging with cardiac motion artefacts. a**) Myocardial signal loss (arrow) due to incomplete re-inversion of the myocardial magnetisation. **b**) Partial blood signal (arrow) due to incorrect inversion time of the blood magnetisation. **c**) Good blood signal nulling, without loss of myocardial signal. In this last example the inversion pulse thickness and timing were optimal for darkening the blood signal.

The inversion pulse preparations used in dark-blood imaging or LGE are particularly sensitive to cardiac motion and arrhythmias. For the multiple inversion pulse preparation used in dark-blood imaging, a correct cardiac cycle synchronisation with the readout is important. If during image acquisition, the heart is not in the same position as when the double-inversion pulses were applied then the myocardial signal can be affected. For example if the inversion pulses occur during systole or early-diastole and the image readout occurs during late diastole then the myocardial wall signal destined to be in the resultant image-slice might be at least partially suppressed by not having been entirely re-inverted by the spatially-selective inversion pulse (Figure 21a). To reduce the potential for this the spatially selective inversion pulse thickness is commonly bigger than the image-slice thickness by a factor of two or three. The trade-off is the re-inversion of blood outside the image-slice potentially reducing the blood signal nulling efficiency for slow flow, which may be a factor for patients with an abnormally low cardiac function. Motion-tracking and offsetting the preparation pulses from the imaging slice have been shown to significantly improve image quality, especially in basal slices (high longitudinal displacements) without the need to increase the slice thickness [53]. Another reason for reduced blood signal nulling efficiency would be an inversion time that was either too long or too short (Figure 21b). Although it is not always possible to change all the parameters required, it is normally possible to adjust the trigger delay and inversion time to change the timings of the preparation and imaging. It should be noted that the terminology for these timing parameters vary from one manufacturer to another.

**Figure 22:**
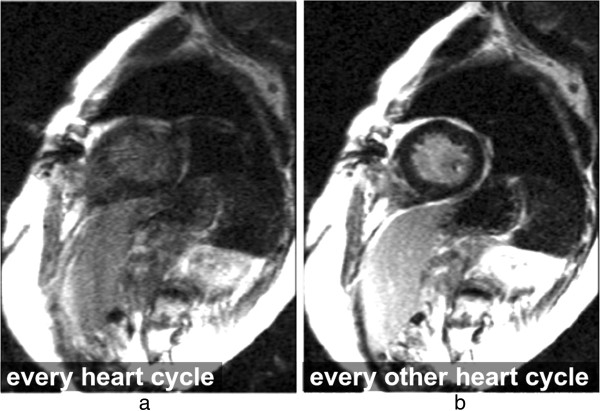
**Arrhythmia artefacts in LGE.** LGE short-axis image, in a patient with arrhythmia. **a**) Image acquisition for every heart-cycle. **b**) Image acquisition for every other heart-cycle. Image quality is improved in *b* by acquiring data at every other heart-cycle only, reducing the contrast inconsistencies between different k-space segments that were created by the irregular heart-rate.

Arrhythmia in LGE can lead to poor image quality due to contrast inconsistencies between different k-space segments, due to variations in the TR for the inversion recovery sequence, thus leading to different amounts of recovery and therefore different levels of magnetisation before and after the inversion pulse. For this reason data is usually acquired for every other heart-beat, reducing dependency on a regular heart cycle but increasing imaging time (Figure 22). For patients with very fast heart-rates it might be required to trigger every 3 heart-cycles in order to guarantee good image quality.

**Figure 23:**
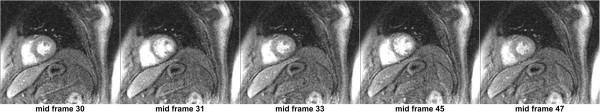
**ECG miss-triggering in myocardial perfusion.** Mid-short-axis slice during a perfusion series. For some frames (31 and 45) the image has been acquired during a different (more rapidly-moving) part of the heart cycle due to ECG miss-triggering.

Poor cardiac triggering can also result in motion artefacts. Most cardiac imaging techniques require the acquisition of full images or k-space segments at specific cardiac phases, i.e. at specific times after the R-wave. Poor triggering due to patients with arrhythmias or weak ECG signal; or due to magneto-hydrodynamic (distortion of the electrical field due to flowing ions in the blood) [54] and/or gradient pulse distortion, can cause effects varying from minor inconsistencies to conspicuous artefacts. For example, poor triggering in myocardial perfusion imaging typically results in slices at different cardiac phases per measurement, this may not degrade the image quality of each individual image but affects the clinical interpretation of first-pass and severely complicates segmentation in quantitative analysis. An example of a perfusion study with miss-triggering is shown in Figure 23.

**Figure 24:**
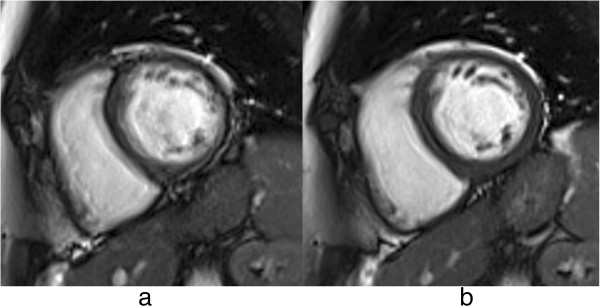
**ECG miss-triggering bSSFP cine.** Short-axis bSSFP cine frame imaged twice: **a**) with significant arrhythmia artefacts and **b**) with reduced artefacts due to a more stable RR-interval.

Triggering problems in cine imaging tend to result in more severe artefacts because each frame’s k-space is segmented across multiple heart-cycles. Mis-triggers causing jumps in TR and or heart-cycle variations will create inconsistencies between k-space segments, thus leading to noticeable cardiac motion artefacts (Figure 24). If ECG triggering quality is low, alternatives can be used such as pulse oximeters, although care has to be taken with the fact that the pulse cycle is delayed from the heart-cycle and not as well defined which reduces timing precision. More recently real-time imaging strategies have been developed where the requirement of ECG triggering and or breath-holding are relaxed [55-59].

### Blood flow

**Figure 25:**
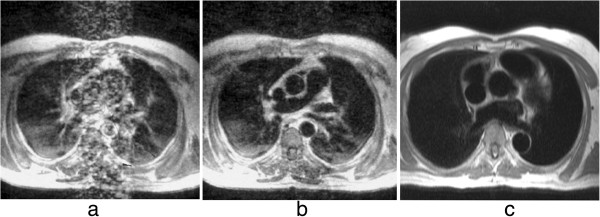
**Spin-echo blood flow artefacts. a**-**b**) Past example of blood flow artefacts on a conventional spin-echo sequence of a transverse slice through the great vessels above the heart: **a**) image acquired in diastole, **b**) image acquired in systole showing reduced flow artefacts due to increased intra-voxel dephasing and time of flight effects. **c**) The introduction of faster segmented acquisitions with black blood preparation avoids this problem, this example shows a similar transverse plane acquired with a HASTE sequence.

**Figure 26:**
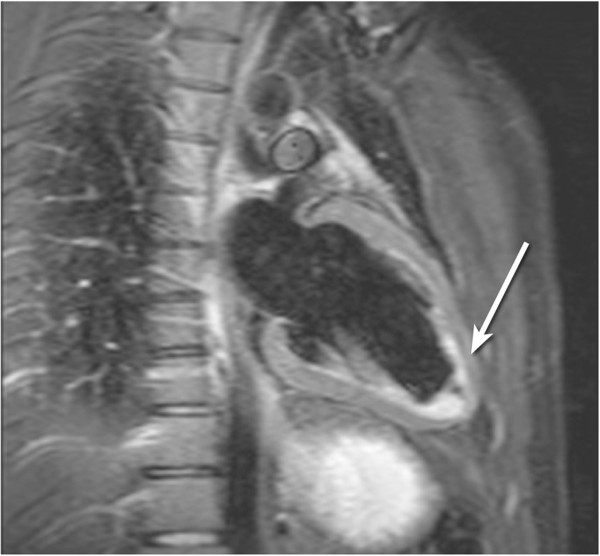
**Residual static blood signal.** STIR image with residual slowly moving blood signal (arrow) that has not been nulled by the double inversion preparation pulse.

Flowing blood has historically been a source of artefacts on CMR images. For conventional sequences the movement of blood results in a signal phase shift related to the velocity of the blood flow. This would not cause a major problem if the blood flow were exactly the same on each successive cardiac cycle of the scan, however, this is not usually the case and the result is that this changing velocity related phase shift will add to and corrupt the spatial phase encoding in such a way that the Fourier transformation will interpret the blood signal as coming from a range of locations spread across the image along the phase-encode direction. To avoid this problem in the early days of CMR for spin-echo acquisitions the images were often acquired in systole at which phase two factors combined to cause a loss of blood signal. Firstly, the blood flow was fast causing increased phase shifts leading to a broad phase distribution at a voxel scale known as intra voxel phase dispersion, and secondly time-of-flight effects meant that the same blood was not excited by both the slice selective 90° and 180° pulses (Figure 25a, b). With the introduction of faster segmented acquisitions of spin-echo images with double inversion blood signal nulling, this problem was largely removed (Figure 25c), although residual static blood at ventricular trabeculations is commonly visible as a white layer in the left ventricle (Figure 26).

**Figure 27:**
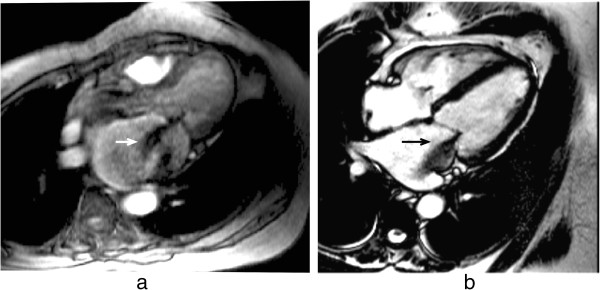
**Complex flow signal loss.** Two examples of a systolic frame of an horizontal long axis cine acquisition from two different patients with insufficient mitral valves: **a**) GRE, **b**) bSSFP. The jet of signal loss caused by complex flows in the left atrium suggests mitral valve regurgitation (arrows). The bSSFP sequence is generally less sensitive to such flow related signal loss.

For gradient-echo acquisitions in the early days the problem was largely removed by the introduction of velocity or flow compensation to null velocity related phase shifts. With the introduction of faster gradient performance and shorter TE’s and consequently greatly reduced velocity related phase shifts, the potential for these artefacts has been reduced, although velocity compensation is still an option to minimise the problem. As a consequence of some cardiovascular diseases, however, the blood flow often becomes much more complex and turbulent and contains higher orders of motion than simple velocity, (acceleration, jerk, etc). These higher orders of flow motion can introduce phase shifts even to a velocity compensated sequence and the spatial scale of this motion is such that this can also lead to significant phase dispersion. This intra-voxel phase dispersion effect is dependent on the TE. Although this signal loss artefact has been found useful by some to assess the severity and form of defective heart valves (Figure 27) it should be used with caution as the area of signal loss may not be directly related to the severity of the valve stenosis.

### Gibbs ringing

Gibbs ringing, also known as a truncation artefact, is present in every unfiltered MRI image and results from the fact that there is only enough time to acquire a finite region of k-space for each image. When the sampled signal is truncated at the k-space edge and then this k-space is inverse Fourier transformed into the image, ringing will unavoidably be present at high-contrast sharp edges of structures on the image. The ringing is a known mathematical limitation of the Fourier transform.

**Figure 28:**
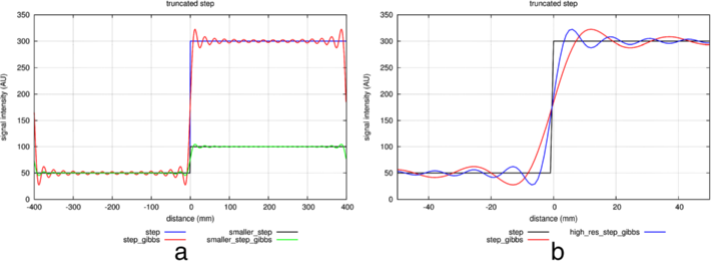
**Gibbs ringing in a signal discontinuity.** Simulations of Gibbs ringing. **a**) Simulations of two theoretical step functions representing an edge on an image with two different signal discontinuities (blue and black). The effects of Gibbs ringing are also shown superimposed (red and green). The higher step discontinuity (blue) will result in more conspicuous ringing (red). **b**) A simulation of a step function (black) sampled with two different spatial-resolutions (red and blue). The frequency of Gibbs ringing scales with resolution, although the amplitude of the under/overshoot is the same for both resolutions.

The two pixels either side of and closest to the edge will show a maximal undershoot and overshoot of the true signal. The magnitude of the under/overshoot can be shown mathematically to be approximately 9% of the edge signal difference; the ringing magnitude is thus dependent on the signal difference at the edge, the higher the signal discontinuity the higher the under/overshoot (Figure 28a). Gibbs ringing also scales with pixel size, i.e. the higher the spatial-resolution the thinner the ringing, but the under/overshoot magnitude does not change with spatial-resolution (Figure 28b). The ringing visibility is also dependent on the edge position inside the pixel [60] (unless zero-filling is applied in k-space for interpolation, when the effect appears consistently).

**Figure 29:**
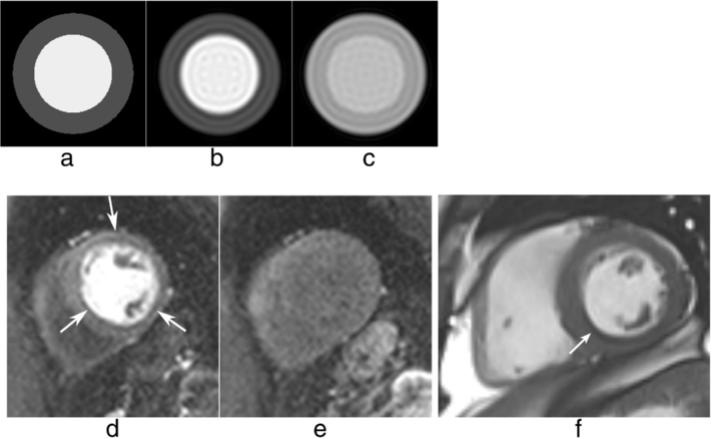
**Gibbs ringing and myocardial perfusion. a**-**b**) Numerical simulation of a short-axis image with an LV/myocardium signal ratio of 3: **a**) theoretical image with no Gibbs ringing, **b**) short-axis image corrupted by Gibbs ringing; notice the darker undershoot in the subendocardial border mimicking a real perfusion defect. **c**) The same short-axis image corrupted by Gibbs ringing but with a lower LV/myocardium contrast to simulate the signal after first-pass; the first Gibbs undershoot is not as dark as in ***b***, and is usually not visible, hidden by the relatively high noise levels of perfusion images. **d**-**e**) *in vivo* short-axis example: **d**) an example of circumferential Gibbs ringing during the first-pass of contrast, **e**) the same short-axis plane after the first-pass when Gibbs ringing is no longer noticeable. **f**) example of Gibbs ringing in a short-axis frame of a bSSFP cine (arrow).

Gibbs ringing is present in all unfiltered MRI images, but it is especially problematic in certain applications such as myocardial first-pass perfusion studies where to reduce acquisition time the spatial-resolution is low. Gibbs ringing can mimic a real subendocardial perfusion defect during first-pass due to the large signal discontinuity between the bright LV blood pool and the darker myocardium [61] (Figure 29d-e). Figure 29f shows an example of suspected Gibbs ringing in a short-axis frame of a bSSFP cine.

One way of reducing Gibbs artefacts is by filtering the k-space data of the image, a process usually known as apodization. Hamming and Hann filters are commonly used in image and signal processing to reduce Gibbs ringing artefacts, although at the penalty of reducing spatial-resolution [62]. Due to the image constraints of most cardiac imaging protocols, any further loss in spatial-resolution is unwanted, and therefore k-space filtering is seldom used due to the fear of reducing diagnostic confidence. It is therefore perhaps better for clinical diagnosis and image interpretation to be done by experts who understand the characteristics of these artefacts and can discriminate between them and true perfusion defects for example. The access to k-space filtering options in the scanner’s protocol may vary for different manufacturers.

Another way of reducing Gibbs ringing is by increasing the spatial-resolution. This will not reduce the ringing magnitude but it will make it less conspicuous due its property of scaling with pixel size. Unfortunately in many cases it is not possible to increase the resolution as this would require increased time, which might not be available. Additionally if it were possible, other sources of artefacts such as cardiac motion could be worsened. However as techniques such as parallel imaging and other image acceleration methods improve they can be used to increase spatial-resolution without increasing imaging time [63-65].

Even though this section focused on myocardial perfusion, Gibbs ringing is visible at any sharp edge, and therefore it could affect many other cardiac applications.

### Aliasing or wraparound artefacts

**Figure 30:**
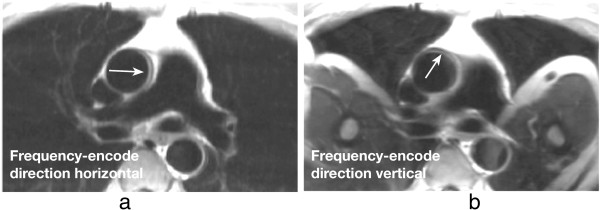
**TSE chemical shift artefacts. a**-**b**) TSE image of a transverse plane through the great vessels above the heart, illustrating chemical shift artefacts between the aortic wall and surrounding fat: **a**) horizontal frequency-encode, **b**) vertical frequency-encode. Artefacts are visible in the aortic wall along the frequency-encode direction (arrows). This artefact can potentially be misdiagnosed as an aortic dissection in some cases. On image ***b*** with the frequency-encode direction swapped from ***a***, wraparound artefacts of the patient’s arms into the chest are also visible.

One of the most basic artefacts commonly seen in MRI is the phase-encode FOV wraparound artefact also known as aliasing. This artefact occurs whenever the size of the object being imaged exceeds the FOV in the phase-encode direction, the outside regions are wrapped into the opposite edge of the FOV. This is due to the failing of the Nyquist sampling requirements of the k-space signal for parts of the object outside the FOV. Aliasing is prevented along the frequency-encode direction either by filtering the frequencies above a limit determined by the Nyquist sampling requirement; or by signal oversampling, i.e. sampling more points in k-space than the ones prescribed by the protocol such that the FOV along the frequency-encode direction is bigger than that actually displayed on the image. The same technique cannot be used along the phase-encode direction without increasing the time of imaging. Therefore this type of artefact is common along the phase-encode direction and also along the slice direction in a 3D acquisition. Figure 30b) that is used to illustrate the chemical shift effect below also very nicely illustrates aliasing of patient’s arms into their chest.

Due to the need of large FOVs of image planes to cover the chest, aliasing is a common problem. If the region of interest is small, for example the heart only, then some wraparound can be acceptable as long as it does not superimpose on the heart. This keeps imaging time short without sacrificing diagnosis and experienced technologists commonly make careful use of this approach.

Another technique to avoid or attenuate this artefact is to use saturation bands (spatially selective saturation pulse as described earlier in the Saturation Pulse section under Cardiovascular Pulse Sequences) in the regions outside the FOV to suppress their signal. The saturation works well only if the signal used for the acquisition is excited only once and immediately after the saturation, such as in the 90° excitation of the TSE sequence.

Aliasing artefacts have been discussed in this section for Cartesian sampling only. Aliasing and undersampling artefacts are discussed further in the *Artefacts Specific to Advanced Cardiac Imaging Methods* section, particularly regarding non-cartesian trajectories and parallel imaging.

### Chemical shift

**Figure 31:**
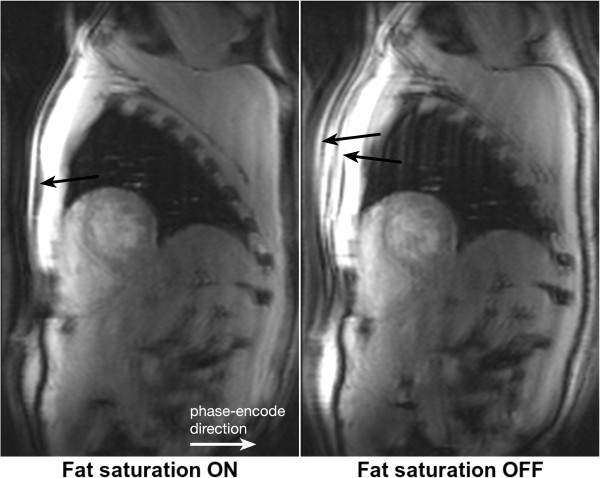
**h-EPI chemical shift artefact.** Short-axis images acquired with a centric interleaved h-EPI sequence with fat-saturation preparation turned on and off. When fat saturation pulses are used, the fat signal in the chest wall is efficiently suppressed (arrow). When there is no fat-saturation preparation, the fat signal in the chest wall is visible and, due to chemical shift, is displaced approximately 4 pixels in both directions (arrows).

The resonance frequency of water and fat differs by approximately 210 Hz at 1.5 T (420 Hz at 3 T), which will result in a number of effects. Firstly a misregistration between fat and water based tissues along the frequency-encode direction and more so along the perpendicular phase blip direction for EPI sequences. Secondly it will result in a slice excitation offset between water and fat; and finally, for gradient-echo sequences only, a possible pixel cancellation effect at water-fat boundaries. The misresgistration in pixels along the frequency direction results from the fact that fat and water that are physically in the same position have different frequencies which will result in the frequency encoding process separating them on the reconstructed image. The distance of separation will depend on the receiver bandwidth. For example, if the bandwidth was 105 Hz/pixel then at 1.5 T their separation of 210 Hz would equate to 2 pixels on the reconstructed image. If the bandwidth were doubled to 210 Hz then the separation would be halved to 1 pixel. Clearly the chemical shift effect is reduced by increasing the bandwidth. This advantage, however, has to be balanced against the loss of SNR when increasing the bandwidth. In most cardiac sequences, misregistration along the frequency-encode direction is relatively negligible, because of the use of large bandwidths for rapid sampling. However, for TSE sequences where longer readouts with lower bandwidths can be used, it is possible to see the effect (Figure 30). As mentioned above, for an EPI readout, misregistration can be a problem and can cause a considerable relative shift of the fat signal along the direction perpendicular to the frequency encoding. For a single shot EPI acquisition the shift can be several pixels and for a typical perfusion h-EPI sequence a chemical shift of close to a pixel is typical. Where centric phase encoding is used, as is often the case, the shift is doubled and split into two opposite directions producing a complicated artefact when fat saturation is not applied correctly (Figure 31). Using a small number of echoes per readout train and fat saturation pulses are common techniques to reduce chemical shift artefacts in EPI.

A fat and water separated imaging technique has been recently published as a method of detecting intramyocardial fat [66].

**Figure 32:**
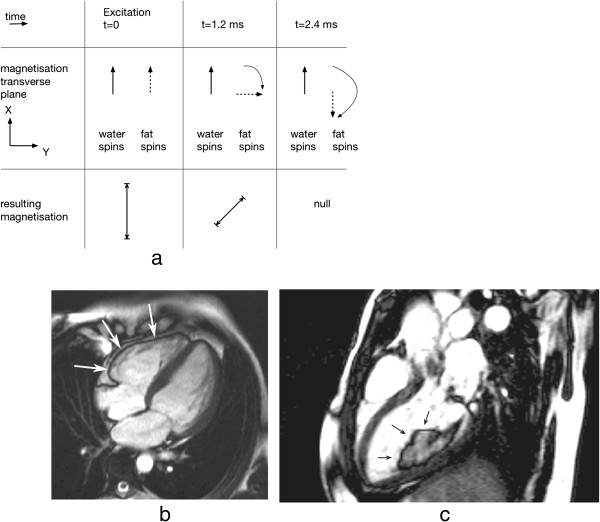
**Fat-water signal cancellation. a**) Diagram showing the evolution of the transverse magnetisation component for a fat spin relative to a water spin. At approximately 2.4 ms at 1.5 T (1.2 ms at 3 T), the spins are out of phase and the resulting signals subtract. **b**) Fat water cancellation artefact around the right ventricular wall (arrows) on a bSSFP long-axis cine. **c**) The same artefact around a liposarcoma in the left ventricle (arrows).

Another important artefact resulting from the chemical shift and sometimes referred to as the *Indian ink* artefact, is the pixel cancellation at boundaries between fat and water based tissues. This occurs with gradient-echo sequences and is TE dependent such that the maximum cancellation occurs when fat-water signals are completely out of phase, which first occurs at a TE of approximately 2.4 ms for 1.5 T (Figure 32). This is often prominent on bSSFP sequence images where the TR has to be kept short enough to minimise the sensitivity of the sequence to field inhomogeneities and this generally leads to a TE in the order of 2 ms (Figure 32b,c).

When imaging the coronaries, the signal cancellation artefacts can reduce the apparent diameter of the coronary arteries if fat signal is not properly suppressed.

### B0-field inhommogeneities

The magnetic field is never completely homogeneous over the volume of the heart. It is possible to largely correct variations by obtaining 3D field plots and calculating the required shim currents, however, local field variations will remain because of the magnetic field susceptibility variations around the heart.

Most tissues are *diamagnetic*, i.e. create a magnetic field that slightly opposes the applied magnetic field. It is important to understand that this arises from their electronic (“Lenz”) diamagnetism, which is much larger than the nuclear magnetisation we employ for MRI. The differences in diamagnetism cause distortion of the main field at interfaces between tissues, and particularly those between tissue and air such as between the heart and lungs. These B0 field inhomogeneities cause resonance frequency offsets, where the local resonance frequency deviates from the scanner’s centre frequency, leading to off-resonance effects. Depending on the local geometry, and the sequence being used, this field distortion may sometimes be more intense causing artefacts such as signal loss or spatial distortion.

**Figure 33:**
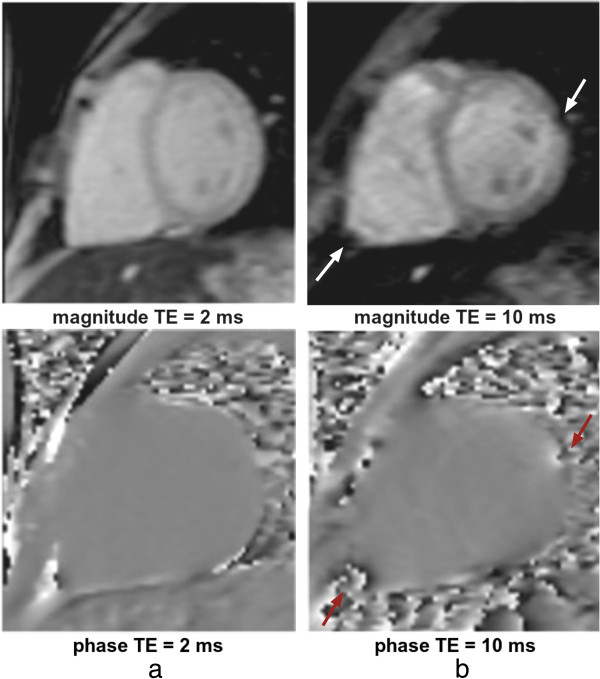
**GRE B0 inhomogeneities at different TEs.** Short-axis images acquired with a GRE sequence at two different TEs (**a**) 2 ms and **b**) 10 ms). Both magnitude and phase images are shown. The arrows point at two local field distortions (RV apex of the heart and in the proximity of a pulmonary vessel) that generate signal loss in the surrounding region as TE becomes larger.

In CMR the sequences with the highest sensitivity to magnetic field inhomogeneities are the bSSFP sequence and sequences that employ rapid k-space acquisition techniques such as EPI and Spiral. The spin-echo sequence is relatively insensitive whilst the conventional gradient-echo sequence has sensitivity depending largely on its TE parameter. Figure 33 illustrates the difference between a TE of 2 and 10 ms for distortion, signal loss, and phase shift caused by susceptibility variations around a pulmonary vessel and around the apex of the right ventricle on a GRE sequence.

**Figure 34:**
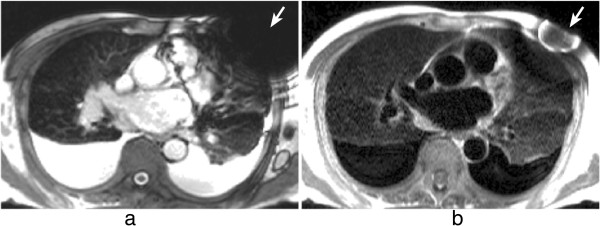
**B0 inhomogeneities and medical devices.** Artefacts caused by an insertable cardiac monitor in a transverse plane through the great vessels above the heart: **a**) image acquired with a bSSFP sequence with visible signal loss and banding artefacts (arrow), **b**) image acquired with a HASTE sequence with less pronounced artefacts (arrow).

**Figure 35:**
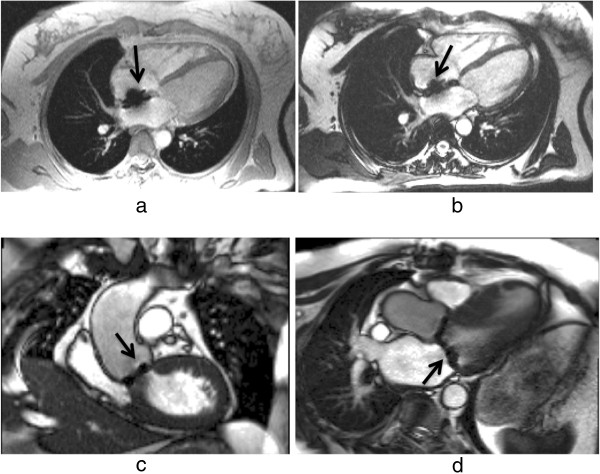
**B0 inhomogeneities and medical devices II. a**-**b**) Four-chamber view acquired with GRE and bSSFP sequences respectively. The arrows point to signal loss caused by a septal occluder. **c-d**) bSSFP image planes containing localised signal loss (arrows) caused by bileaflet aortic and mitral valve replacements respectively.

**Figure 36:**
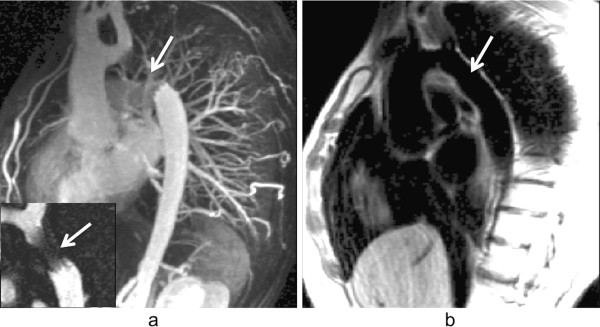
**B0 inhomogeneities and medical devices III. a**) 3D GRE contrast-angiography maximum intensity projection reconstruction image showing signal loss in the descending aorta due to an aortic stent (arrow), the bottom left corner shows a bSSFP image plane containing the aortic stent; also showing signal loss in the same region (arrow). The signal void inside the stent is likely to be due to lack of RF penetration through the stent wall. **b**) Spin-echo black-blood image also in a plane containing the aortic stent. The aortic wall outside the stent is clearly seen (arrow).

Figure 34 illustrates an example where a patient with an insertable cardiac monitor has been scanned with both bSSFP and HASTE sequences. The bSSFP image shows signal loss and banding artefacts in the region proximal to the device due to magnetic field distortions, while the HASTE sequence is less sensitive. Many other medical devices are also a source of susceptibility artefacts by distorting the magnetic field (Figure 35). Figure 36 shows an example of a patient with an aortic stent that will cause localised field inhomogeneities and signal loss on the gradient echo images used for the 3D MR angiogram. In this case, however, there is also an RF shielding effect of the stent blocking RF pulse penetration, contributing to the lack of signal from the region of the stent lumen.

**Figure 37:**
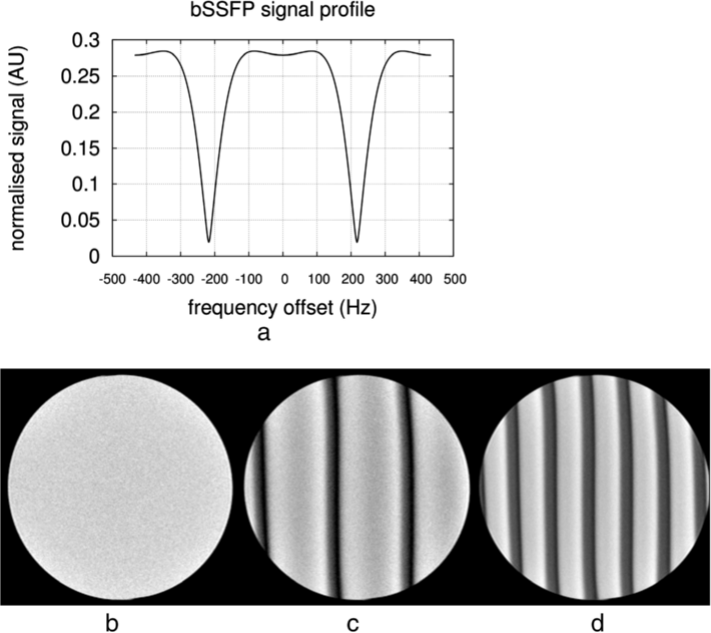
**bSSFP and B0 inhomogeneities. a**) bSSFP steady-state signal as a function of frequency-offsets (RF flip angle 50 degrees, TR 2.2 ms). The signal is approximately constant except at multiples of ≈±227 Hz where the signal drops down to near zero, forming dark bands in these regions. **b**) Spherical phantom imaged with a bSFFP sequence with an homogenous B0. **c**) Phantom image repeated with a field gradient from left to right. Dark bands are formed where the frequency offsets are multiples of ≈±227 Hz. **d**) Similar to *c* but with a TR twice as large, halving the distance between dark bands as a result.

The bSSFP sequence is commonly used in functional analysis and other applications. For this pulse sequence, B0 inhomogeneity artefacts are very distinct and can be very severe. The steady-state signal response to B0 inhomogeneity is plotted in Figure 37a, which shows the available signal as a function of frequency-offset. The signal remains approximately constant for small frequency-offsets, but drops down to near zero to form dark bands at regularly-spaced frequency offsets [67]. The more inhomogeneous the magnetic field, the larger the frequency distribution in the image volume and the more black bands will appear. The frequency separation between regions with no signal is inversely proportional to the TR of the sequence; thus, reducing the TR reduces the number of artefacts observed (Figure 37c-d).

Localised field inhomogeneities due to susceptibility differences in the locality of the cardiac veins [68]; and at tissue-air interfaces such as the heart-lung interface [69], or adjacent to air pockets [70] (in the bowel, stomach, and colon for example), introduce banding type artefacts on bSSFP images that can confuse the image interpretation. These signal loss artefacts should not be confused with intravoxel dephasing which is caused very differently.

**Figure 38:**
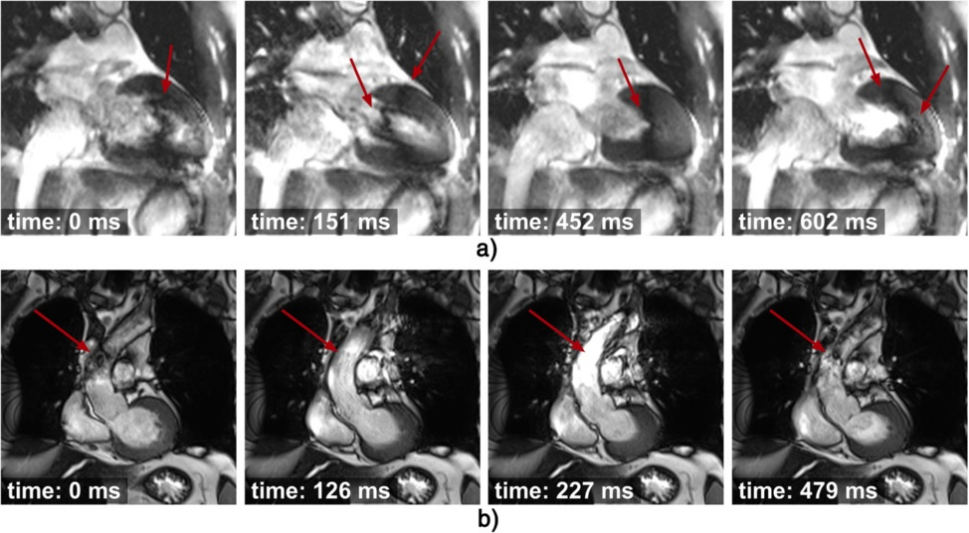
**bSSFP and B0 inhomogeneities: blood flow and dark bands.** Two bSSFP cines with two examples of off-resonance artefacts and the effects of blood flow, the acquisition time after the R wave is also shown for each image. **a**) A dark band is visible in the heart (arrows), its position varies throughout the heart cycle due to myocardial motion and blood flow. **b**) Dark banding and signal intensity variations are visible in the ascending aorta (arrows) due to a dark band in the proximity of the image plane. Its effects are brought into the image plane by the flowing blood in the ascending aorta.

In the bSSFP sequence, blood flow can also create artefacts in the presence of field inhomogeneities. Moving spins can exhibit signal variations and loss due to the breakdown of the steady-state signal [71-73]. These can occur due to in-plane flow moving through an off-resonance region, but also for through-plane flow leaving the image plane into such a region (Figure 38).

**Figure 39:**
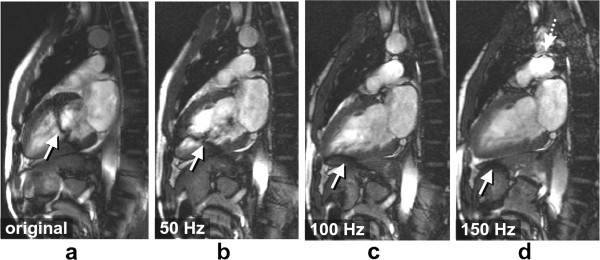
**bSSFP and B0 inhomogeneities: tuning frequency. a**-**d**) Series of vertical long axis bSSFP images acquired with different tuning frequency offsets: original (0Hz), 50Hz, 100Hz, and 150Hz. Black band and flow artefacts through the ventricle are shown (arrows). As the frequency is adjusted the artefacts are shifted away from the heart. Image ***d*** shows another dark band approaching the heart from the top (dotted arrow), as the tuning frequency is shifted. This approaching frequency-offset causes the blood signal to be hyperintense in the image plane, similar to that shown in Figure 38b) (227 ms).

**Figure 40:**
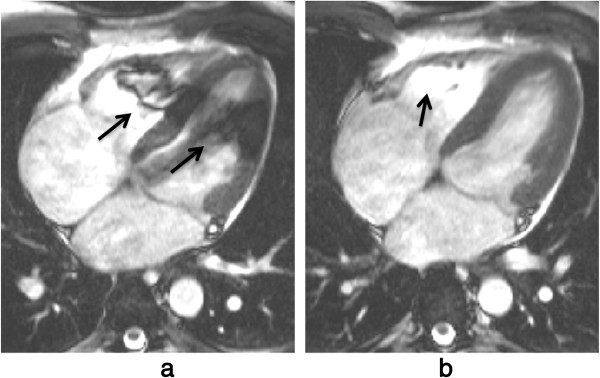
**bSSFP and B0 inhomogeneities: tuning frequency II. a**) Long-axis bSSFP cine frame showing dark bands in the heart (arrows) due to field distortions caused by an air pocket in the colon (not shown in this plane). **b**) The same frame after a manual frequency tuning. The shift of the scanner centre-frequency removed the artefact from the LV, although signal enhancement artefacts due to blood flow from the image plane into a dark band region are still visible in the RV (arrow).

**Figure 41:**
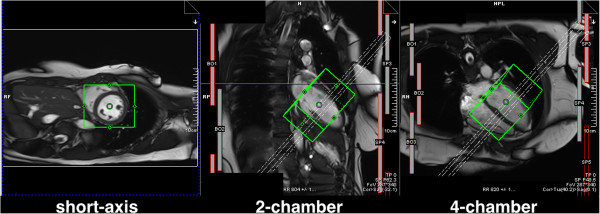
**Frequency and shim adjustment box.** Example of the positioning of the frequency and shim adjustment volume (represented by the green box) shown in three anatomical slices. The dashed rectangle represents the position of the short-axis slices being acquired. The appearance and application of this will vary between manufacturers.

More dark bands and signal variation artefact are displayed in Figure 39. If a black band or flow in the presence of field inhomogeneities artefacts affect an image then it is possible to move and or suppress these by reacquiring with a slight frequency offset (≈ 50Hz) of the scanner centre-frequency. It should be recognised, however, that as one black band moves out of the heart then another may be moving in from the other side (Figure 39d). Another example of frequency retuning is shown in Figure 40 for a patient with an air pocket in the colon. Artefacts can thus be reduced by better shimming of the field in and around the heart and with localised centre-frequency adjustments. It is also sometimes crucial with such localised adjustments to understand that they are not confined to the slice thickness; in the situation where through-plane flow or motion is large, then the shim adjustment should include sufficient distance perpendicular to the plane to allow it to obtain homogeneous field volume around the slice (Figure 41). Another technique known as wideband bSSFP [74] can also be used to reduce the dark band artefact, although with increasing scanning time.

Field inhomogeneities are also an important source of artefacts for the less widely-used EPI sequences. Sequences with an EPI readout tend to be used in a hybrid approach (h-EPI), in order to keep the readout train reasonably short, thus minimising blurring, ghosting, image distortion, and chemical shift artefacts. h-EPI sequences are commonly used with an interleaved phase-order. Frequency offsets will introduce phase variations in a stepwise fashion, which can lead to artefacts such as blurring, and ghosting.

**Figure 42:**
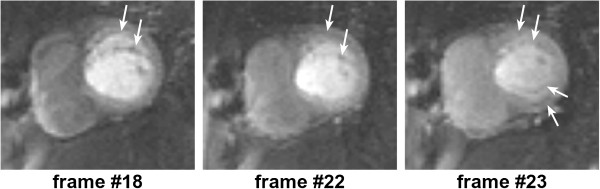
**h-EPI perfusion and B0 inhomogeneities.** Three frames during contrast first-pass perfusion imaging in a basal short-axis plane acquired with an h-EPI sequence. This example shows severe frequency-offsets which result in blurring and ghosting (arrows) along the phase encode direction (vertical) with this sequence. This example is extreme and uncommon, although blurring of the myocardial wall along the phase-encode direction is believed to be a more common event in first-pass perfusion with this particular sequence.

Magnetic field inhomogeneities caused by the arrival of a strong paramagnetic susceptibility contrast agent in the heart are commonly attributed as a possible mechanism for dark rim artefacts in myocardial perfusion imaging. The frequency offsets introduced in the myocardium by the first-pass of a Gadolinium-based contrast agent have been measured at rest and shown to be insignificant for intra-voxel signal dephasing in the myocardium at 1.5 T [75], although when combined with other sources of field inhomogeneities or tuning frequency-offsets it might produce artefacts such as signal loss or image degradation. The perfusion tailored h-EPI sequence has a centric interleaved phase-order [36], which makes it very sensitive to frequency-offsets [76], resulting in ghosting and splitting of the image along the phase-encode direction due to its centric sampling trajectory (Figure 42).

In general to minimise B0-inhomogeneities, careful shimming and especially localised scanner frequency adjustments are advised prior to imaging.

### Blood flow measurement

**Figure 43:**
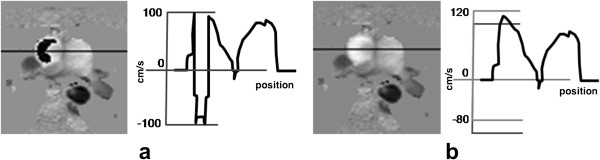
**Blood flow aliasing.** Phase contrast velocity map and velocity profile from a transverse slice showing flow in the great vessels above the heart. **a**) Aliased velocities are seen in the ascending aorta. **b**) Velocity measurement corrected by “offsetting” the velocity window post-scan. The offset shifts the range of velocities corresponding to black and white pixels, in this example upwards so that the fast forward velocity does not exceed the top of the range. Although this “offset” correction usually suffices in simple cases (like this one) where pixels of wrapped velocity are surrounded by non-wrapped pixels, problems can occur in other situations where the wraparound has occurred in pixels at the edge of a flow. This results in “uncorrectable” aliasing where no amount of offset produces a sensible value for the wrapped pixels. This correction is not generally available on scanners, but is available in post image-processing software.

As described earlier, blood flow is measured by phase contrast velocity maps, i.e. pixel maps of phase differences between two reconstructed phase images each acquired with a different phase velocity sensitivity. Because they are a map of phase difference there is an inherent maximum range of phase (360°) and therefore a maximum range of velocity that can be measured. The phase sensitivity of the acquisition sequence can be adjusted according to the expected flow velocities, however, if a larger than expected range of velocities is encountered then the phase range will be greater than ±180° and artefactual or so called aliased velocities would be measured (Figure 43). In general, the chances of aliasing should be minimised by estimating a larger than expected velocity range, but at the same time without compromising the sensitivity of the velocity measurement.

**Figure 44:**
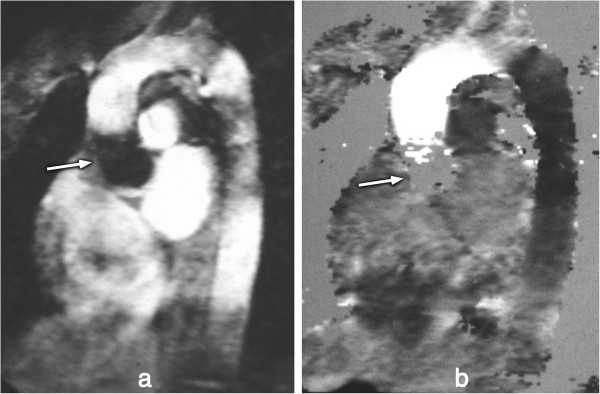
**Blood flow misregistration.** An old study showing an image acquired with a relatively long TE showing zero signal of the blood flowing into the slice (arrow) and misregistration of blood signal along the ascending aorta: **a**) magnitude, **b**) phase. This effect is generally now reduced due to shorter TEs being used, although it could still be a problem for fast moving jets of blood through stenotic valves for example.

There are a number of artefacts and measurement errors that can be encountered with phase contrast velocity mapping. These include artefactual velocity measurements due to aliasing of signal phase as described above. Another problem is that of signal misregistration where blood signals move between the timing of slice selection and phase and frequency encoding (Figure 44). This can result not only in the artefactual positioning of the blood signal relative to the stationary ones but also artefactual velocity measurements due to an overlay and combining of moving and stationary spins and their phases. For similar reasons partial volume effects where a voxel contains a mixture of stationary and flowing spins will result in measurement errors, especially if the flowing spins have velocity aliasing as described above.

More subtle effects can cause significant errors to flow measurements by introducing background phase shifts. Some distortion of the magnetic field gradients is unavoidable from fundamental electromagnetism, and this is known in MRI as *Maxwell* or *concomitant gradients*[77]. This is one cause of the background phase shifts which become more significant when high-strength gradients are used and also when imaging at lower main field strengths. With the latest gradient systems Maxwell gradient effects are certainly a factor for phase contrast velocity mapping at 1.5T. The velocity maps can however be corrected analytically and automatically in software with no user intervention required.

A second common reason for background phase shifts is the presence of small uncorrected side-effects of the gradient pulses in the magnet, known as *eddy currents*. Software is sometimes provided which allows the user to place markers identifying stationary tissues so that the background phase error can be calculated and removed from the entire image. It has been shown previously that small background errors (smaller than 1% of the velocity sensitivity range (VENC)) can significantly affect measurements of shunt flow and cardiac output, and that these may occur easily on some systems [78].

Voxels containing a range of velocities in the direction encoded will suffer dephasing, resulting in a weak overall signal which can lead to unreliable phase measurements and therefore unreliable output from the phase subtraction for the velocity image. As the velocity encoded magnitude is often not reconstructed users should be aware of how unreliable the velocity data may be.

### Late gadolinium enhancement

**Figure 45:**
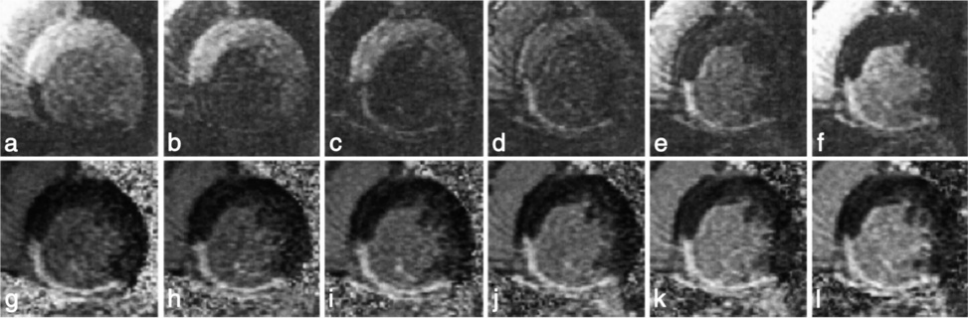
**LGE: TI timing (magnitude vs PSIR).** Short-axis images at varied TIs for a patient with inferior MI. **a**-**f**) Magnitude, **g**-**l**) normalized phase-sensitive. TI = 175, 200, 225, 250, 275, and 300 ms from left to right. The appearance and contrast are variable for the magnitude-reconstructed images, while they are consistent for the normalised phase-sensitive reconstruction (Reprinted, with permission, from reference [38]).

In LGE studies, choosing the correct inversion time (TI) to null normal myocardium can be challenging for inexperienced operators. The time varies from patient to patient and increases with time after the injection; typical inversion times are around 200-300 ms. At the correct TI, normal myocardium is nulled, while non-viable tissue is bright. With the standard magnitude reconstruction, if TI is too short or too long then normal myocardium will not be nulled, and the contrast between infarcted and normal myocardium is highly reduced (Figure 45). To overcome this problem, phase sensitive image reconstruction (PSIR) [38] allows the normal myocardium to be effectively nulled retrospectively whatever the TI by adjusting the image window and level on the display.

**Figure 46:**
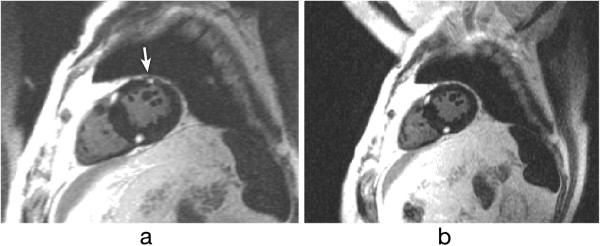
**Phase-swapping in LGE.** Short-axis LGE image with phase-encode direction: **a**) horizontal, **b**) vertical. Artefact of an unknown source can be seen mimicking gadolinium enhancement on image **a** (arrow), but not on **b**.

A potential phase-encode artefact in LGE imaging due to the longitudinal magnetisation inversion recovery during the acquisition of multiple phase-encode lines in each cardiac cycle has been debated [79]. The magnetisation recovers throughout acquisition window from negative to positive with potential consequences to the image. Some centres acquire “phase-encode swapped” repetitions of LGE images, partly to identify any such artefact by its direction rotation with the phase encoding, but also as a safeguard to detect other potential artefacts that direct themselves along the phase-encode direction, such as those caused by respiratory motion,CSF ghosting and other factors (Figure 46).

**Figure 47:**
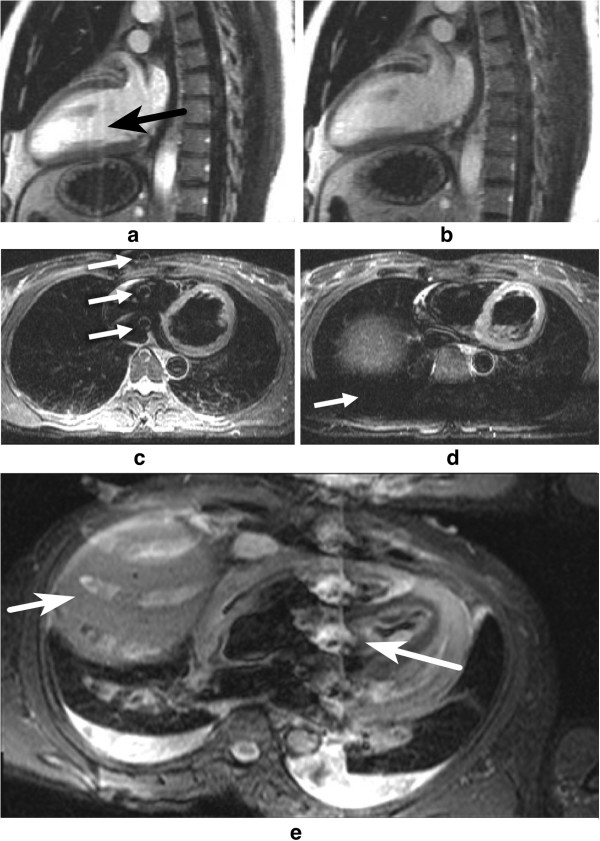
**CSF and pleural effusion ghosting. a**-**b**) Long-axis LGE image: **a**) CSF ghosting is visible in the heart (arrow), **b**) CSF ghosting suppressed by a saturation band placed over the spinal canal. In this example the spatial location of the saturation is not very clear, this is presumably because of the timing of the spatial saturation that cannot immediately precede the imaging k-space centre, and the Mz recovery of other tissues due to gadolinium shortened T1. However, the T1 of CSF remains very long, which means that this stays well saturated. **c**-**d**) STIR transverse image: **c**) CSF ghost visible in the RV (arrows), **d**) CSF ghosting supressed by saturation band placed over the spine (arrow). **e**) Ghosting of the fluid in the pleural layers (pleural effusion) (arrows) in part due to its long T1. Pulsatility effects may also be present, contributing to the ghosting.

One artefact that does not only affect LGE studies but any segmented acquisition with an inversion pulse is the ghosting of fluids with a long T1. The long T1 produces a signal oscillation during its approach to the steady-state (see Gradient-Echo section), where the signal tends to invert the magnetisation sign in alternate cycles of the acquired data resulting in ghosts along the phase-encode direction. Ghosting of the cerebrospinal fluid (CSF) which has a very long T1 is common in LGE and STIR black-blood studies. This is usually suppressed by applying a spatial saturation band (Figure 47a-d). In the LGE example (Figure 46a-b) the spatial location of the saturation is not very clear, this is presumably because of the timing of the spatial saturation that cannot immediately precede the imaging k-space centre and the Mz recovery of other tissues due to gadolinium shortened T1. However, the T1 of CSF remains very long, which means that this stays well saturated. A possible example of the same ghosting mechanism of a pleural effusion in a STIR sequence is also shown in Figure 47e.

### Artefacts specific to advanced cardiac imaging methods

Methods of more efficiently acquiring the MR data are of particular interest to CMR because of the compromises that have to be made to acquire image data in a short time, often restricted to a fraction of one cardiac cycle or fractions of multiple cardiac cycles within a breath-hold. Methods such as non-cartesian sampling, parallel imaging, and partial-Fourier have been introduced to accelerate the acquisition. Although each of these methods may work well a proportion of the time and in particular if extra care is taken to set up the scan, they all tend to reduce the imaging robustness and tend to produce artefacts on images and or produce artefactual measurements.

### Parallel imaging artefacts

Parallel imaging accelerates image acquisition which has the potential to reduce some problems, for example, although parallel imaging introduces an SNR penalty, it also shortens the readout time which for a single shot EPI sequence for example reduces distortions caused by field inhomogeneities, reduces blurring due to T2* and or T2 decay and reduces sensitivity to motion.

**Figure 48:**
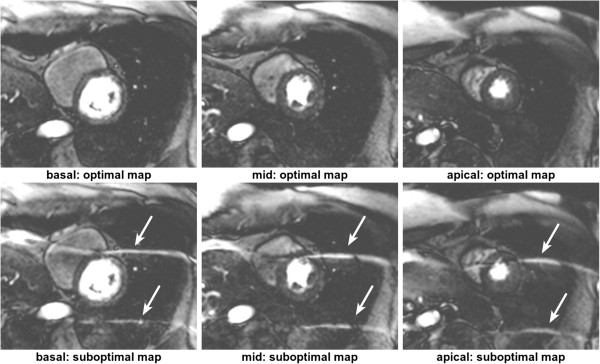
**SENSE with aliasing artefact due to respiratory motion.** Three short-axis slices during first-pass perfusion reconstructed with two different coil sensitivity maps (acquisition with a bSSFP sequence and SENSE with an acceleration factor of 4). The three slices on top were reconstructed with the optimal coil sensitivity map, i.e. the coil sensitivity map acquired in the same respiratory position as the perfusion images. On the bottom the same perfusion images were reconstructed with a coil sensitivity map that was acquired in a different respiratory stage from the perfusion images. The reconstruction is corrupted with aliasing artefacts (arrows) due to inconsistencies between the sensitivity map and the undersampled perfusion data.

SENSE and GRAPPA are two parallel imaging techniques that, in theory, should yield similar results; although in some situations GRAPPA is more robust to artefacts because the coil sensitivity maps are generated in k-space, and not in the image-space as for SENSE. For example, when imaging aliased FOVs, SENSE coil sensitivity maps may also be aliased, and therefore erroneous image reconstruction will result in incomplete unwrapping of the aliased pixels [80] (some scanner manufacturers acquire sensitivity maps over a much larger FOV than the image acquisition in order to avoid this issue). Respiratory motion can also introduce reconstruction errors due to inaccurate sensitivity maps (Figure 48) [81].

### Motion with radial and spiral k-space trajectories

Sequences with non-Cartesian forms of k-space coverage such as radial and spiral have also been used to image the heart [82-88], artefacts discussed earlier can have very different characteristics when compared to the more conventional Cartesian methods.

**Figure 49:**
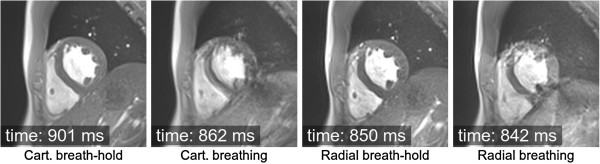
**Impact of respiratory motion on cine imaging: Cartesian vs radial.** Short-axis GRE cine, comparing the effects of respiratory motion on a Cartesian vs Radial k-space acquisition. In both sampling trajectories two different acquisitions were made, one during a breath-hold and another during free breathing. The acquisition time after the R wave for each image is also shown.

Radial and spiral k-space acquisitions are generally thought of as being less sensitive to motion. Radial sequences tend to oversample the centre of k-space and this tends to reduce the visibility of breathing artefacts [89]. For a radial acquisition instead of ghosting in the phase-encode direction as seen with Cartesian sampling, respiratory motion results in streaking artefacts propagating from the moving object, perpendicular to the motion direction (Figure 49). For spiral sequences, motion artefacts tend to take the form of swirls and depending on the imaging region of interest these can be less of a problem than phase-encode ghosts. Variable-density spirals that oversample the centre of k-space, at a cost of undersampling the edges, have also been shown to reduce respiratory artefacts in segmented cine images [83].

Radial sampled data can also be used to correct for motion in a self-navigating way. Different projection views (at different angles) can be analysed for consistency. If motion is present, consistency will fail and the data can be discarded and potentially resampled before reconstruction [90]. This technique uses navigator data from the heart itself, and not from the diaphragm, eliminating any problems of translating diaphragmatic motion to cardiac motion. Similarly self-gating has been described using radial sampling with a 1DFT projection reconstruction to monitor the heart motion through the cardiac cycle and then to use this for cardiac gating [91]. However, it has to be accepted that the performance of such methods depends on the contents of the slice being imaged and is potentially less reliable than the use of ECG synchronisation.

### B0 inhomogeneity with Radial and Spiral k-space trajectories

**Figure 50:**
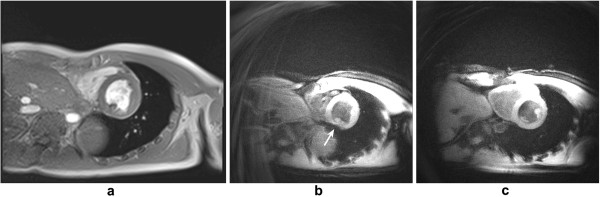
**Off-resonance and spiral sampling. a**) Cartesian mid-ventricular short-axis slice cine frame, this image is shown for morphological comparison with image ***b***. **b-c**) Spiral magnitude velocity map (with a black-blood preparation) frames at mid and basal slice planes respectively. A region of off-resonance in the inferior myocardial wall (arrow) causes signal loss artefacts in the mid-ventricular slice plane. This artefact is less visible in the basal slice.

Non-Cartesian approaches with long (>15 ms at 1.5 T) acquisitions after each RF pulse are highly sensitive to field inhomogeneities, where generally off-resonance and chemical shift translates into image blurring in all directions and signal loss (Figure 50). In spiral trajectories, blurring increases with increase in readout time of one interleave, i.e. with the off-resonance phase accumulated along each readout. Radial trajectories show geometrical and image intensity distortions in the presence of field inhomogeneities [92]. B0-inhomogeneity typically varies slowly over the FOV, whereas magnetic susceptibility can vary sharply, resulting in spatially dependent blurring and signal loss.

### Aliasing with radial and spiral k-space trajectories

Due to the 2D readout gradients in these trajectories, aliasing artefacts happen from all directions resulting in additional streaks and swirls, therefore the FOV has to be chosen with attention to aliasing in all directions. Because there is no wraparound along the phase-encode direction, mild undersampling artefacts are commonly considered as a good trade-off for reduced imaging time.

### EPI and nyquist ghosting

**Figure 51:**
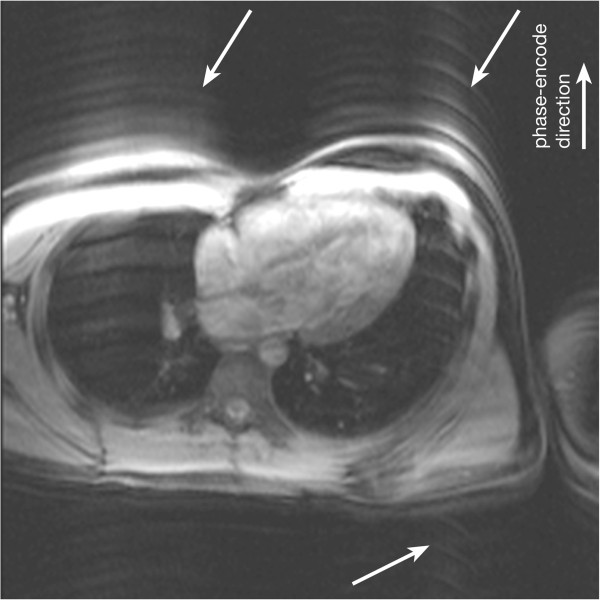
**Nyquist ghosting: interleaved h-EPI sequence.** Long-axis image acquired with an interleaved h-EPI sequence during a breath-hold. The arrows point to the multiple Nyquist ghosts that spread along the phase-encode direction.

The readout of an EPI echo train also introduces a specific artefact related to the alternating sampling gradients. This artefact is commonly known as *Nyquist ghost* or *N/2 artefact* and it is created by a mixture of gradient imperfections [93], eddy currents, concomitant fields [94], receiver filter asymmetry [95] or susceptibility. All these factors will cause mismatches between odd and even echoes, resulting in some signal or a “ghost” being displaced half a field of view across the image along the phase-encode direction. These mismatches can be measured by acquiring at least two non-phase-encoded echoes with opposite polarity during imaging. The acquired mismatch can then be corrected in a post-processing technique known as phase-correction, reducing the visibility of Nyquist ghosting. This correction is commonly employed in EPI sequences. When using h-EPI techniques with multiple excitations per acquisition, then the phase discrepancy between odd and even lines leads to multiple ghosts [96]. Figure 51 shows an example of an h-EPI image with imperfect phase-correction, displaying residual Nyquist ghosting along the phase-encode direction.

### 1.5T vs 3T

Cardiac imaging at 3T is becoming increasingly popular due to the potentially higher SNR and CNR (Contrast to Noise Ratio). At higher fields, some of the above mentioned artefacts become even more problematic such as those caused by B0 and B1 field inhomogeneities or chemical shift. The potential increase in SNR, however, can be traded for example for higher parallel imaging accelerations resulting in quicker imaging acquisitions, which can potentially reduce motion artefacts; or enable higher spatial-resolutions which can for example make Gibbs artefacts less prominent.

Together with an increase of approximately twofold in SNR, there is also a quadruple increase in RF power absorption. Some sequences, such as bSSFP, may have to be used with a reduced flip-angle and higher TR than at 1.5T, which increases B0 inhomogeneity sensitivity and reduces contrast. An experienced operator with the ability to perform careful frequency adjustments is important when using bSSFP sequences at 3T. EPI geometric distortions and ghosting are also a bigger problem at a higher field. Spoiled GRE sequences are thus more common in many 3T imaging protocols, taking advantage of the spoiled sequence’s increased robustness to B0 inhomogeneities along with the increased SNR over 1.5T.

Although T2 relaxation times of tissues remain approximately unchanged from those at 1.5T, T1 relaxation times are noticeably longer at 3T [97]. This requires many protocols to be adapted for use at 3T. For example the optimal inversion times for black blood imaging and late-Gd-enhancement may need to be increased. The same changes in relaxation time can also be used to reduce the dose of Gd contrast agent in first-pass perfusion and LGE studies decreasing possible susceptibility problems [98,99].

Cardiac gating can also be more challenging at 3T. The higher field strength creates more magnetohydrodynamic distortion of the ECG signal, which can prevent the scanner from gating properly leading to additional artefacts.

## Conclusion

Cardiovascular imaging is complicated primarily by the complex nature of the cardiac motion. Many of the cardiac imaging artefacts are directly related to motion or indirectly introduced by the requirement to shorten the acquisition time to remove motion. The complex cardiac structure with mixtures of fat and water based tissues containing complex and varying blood flows, and the large chest region with many organs and tissue-air interfaces also open the door to additional artefacts and measurement errors.

If we understand the physical principles behind the formation of artefacts, we should be in a position to identify and possibly avoid them, increasing image quality and interpretation.

## Competing interests

The authors declare that they have no competing interests.

## Authors’ contributions

All authors contributed in the design, intellectual conception, and revision. All authors read and approved the final manuscript.
